# C19-Norditerpenoid Alkaloids from *Aconitum szechenyianum*

**DOI:** 10.3390/molecules23051108

**Published:** 2018-05-08

**Authors:** Bei Song, Bingliang Jin, Yuze Li, Fei Wang, Yifu Yang, Yuwen Cui, Xiaomei Song, Zhenggang Yue, Jianli Liu

**Affiliations:** 1 The College of Life Sciences, Northwest University, Xi’an 710069, China; songbei168@126.com (B.S.); lyz1990yeah@163.com (Y.L.); 2 Shaanxi Collaborative Innovation Center of Chinese Medicinal Resource Industrialization, School of Pharmacy, Shaanxi University of Chinese Medicine, Xianyang 712046, China; songxiaom@126.com; 3 Experiment Center for Science and Technology, Shanghai University of Traditional Chinese Medicine, Shanghai 201203, China; jin872459317@126.com (B.J.); yangyifu@mail.shcnc.ac.cn (Y.Y.); 4 Shaanxi Institute for Food and Drug Control, Xi’an 710065, China; wf88-88@163.com; 5 Department of Pharmacy, Xi’an Medical University, Xi’an 710021, China; polaris_101025@163.com

**Keywords:** *Aconitum szechenyianum*, C_19_-norditerpenoid alkaloids, immunosuppressive effects

## Abstract

Three new C_19_-norditerpenoid alkaloids (**1–3**), along with two known C_19_-norditerpenoid alkaloids (**4,5**), have been isolated from *Aconitum szechenyianum*. Based on extensive spectroscopic techniques (1D, 2D-NMR, IR, and MS) and chemical methods, their structures were established as szechenyianine D **(1)**, szechenyianine E (**2**), szechenyianine F (**3**), 8-*O*-methyl-14-benzoylaconine (**4**), and spicatine A (**5**). The immunosuppressive effects of compounds **1**–**5** were studied using a ConA-induced or LPS-induced splenocyte proliferation model. In vitro tests showed that Compounds **2, 4**, and **5** suppressed ConA-induced or LPS-induced splenocyte proliferation in a concentration-dependent manner. The CC_50_/IC_50_ values of **2, 4,** and **5** suggested that these compounds were potential immunosuppressive agents for the treatment of autoimmune diseases characterized by arthritis, such as rheumatoid arthritis.

## 1. Introduction

**Figure 1:**
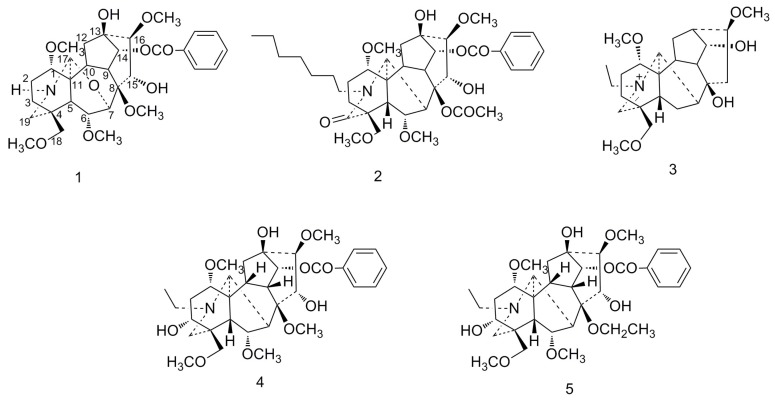
Structures of Compounds **1**–**5**.

The roots of *Aconitum szechenyianum* Gay. and *A*. *flavum* Hand.-Mazz., which belong to the *Aconitum* genus of Ranunculaceae, are widely used in folk medicine in Shaanxi province in China [1]. C_19_- and C_20_-diterpenoid alkaloids possessing aconitine-type, 7,17-secoaconitine-type, and napeline-type skeletons, which are the main components of *A*. *szechenyianum* [2,3,4,5], possess anti-inflammatory, analgesic, anticancer, anti-epileptiform, and antiparasitic activities [6,7,8]. *A. szechenyianum* has been preliminarily studied; in that study, several norditerpenoid alkaloids were obtained with an aconitine or 7,17-secoaconitine skeleton, and these skeleton were demonstrated to have anti-inflammatory activities in a dose-dependent manner [9]. This paper reports a new investigation on *A. szechenyianum*, conducted to explore more bioactive lead compounds, and three new, along with two known, C_19_-norditerpenoid alkaloids—szechenyianine D (**1**)*,* E (**2**), F (**3**), 8-*O*-methyl-14-benzoylaconine [10] (**4**), and spicatine A [11] (**5**)—were isolated in different fractions from the previous study (Figure 1). The previous study shows that the active ingredients of *A. flavum* exhibit immunosuppressive effects [12,13], which had not been previously reported from *A. szechenyianum.* Therefore, the immunosuppressive effects of Compounds **1–5** were evaluated in vitro through ConA- or LPS-induced splenocyte proliferation models. Three compounds inhibited ConA- or LPS-induced splenocyte proliferation, revealing for the first time that the roots of *A. szechenyianum* possess immunosuppressive activities.

## 2. Results

**Figure 2:**
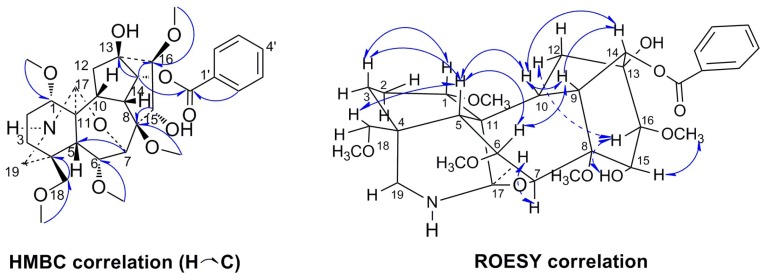
Key HMBC (H→C) and ROESY (H↔H) correlations of Compound **1**.

Szechenyianine D (**1**) was isolated as a white amorphous powder and showed a positive reaction with Dragendorff′s reagent. Its molecular formula C_31_H_43_NO_10_ was derived from the protonated molecular ion peak at *m/z* 590.2979 [M + H]^+^ (calcd. 590.2965) in the HR-ESI-MS spectrum. The ^1^H-NMR spectrum (Table 1) of **1** showed the presence of five aromatic proton signals due to a monosubstituted benzene at δ_H_ 8.00 (2H, d, *J* = 7.4 Hz), 7.54 (1H, t, *J* = 7.4 Hz), and 7.44 (2H, t, *J* = 7.4 Hz), five OMe protons at δ_H_ 3.80(3H, s), 3.37 (3H, s), 3.29 (over-lapped), 3.29 (over-lapped), and 3.21 (3H, s), and two strongly shielded protons at δ_H_ 3.32 (1H, s) and δ_H_ 4.29 (1H, s). The ^13^C-NMR spectrum (Table 1) displayed 31 carbon resonances. Among them, resonances at δ_C_ 166.4, 133.4, 130.0, 130.0 (C × 2), and 128.7 (C × 2) were attributed to a benzoyloxy group; δ_C_ 62.2, 59.3, 59.0, 55.4, and 50.7 were attributed to five OMe groups; δ_C_ 74.7 and 76.4 were attributed to two oxygenated carbons associated with hydroxyl groups. Out of the 10 oxygen atoms in **1**, **9** are associated with five methoxy groups, two hydroxyl groups, and one benzoyl group, and the remaining one may be a hydroxyl group or an internal ether. The NMR features of the remaining 19 resonances were characteristic of an aconitine-type alkaloid, where the δ_C_ 64.4 and 70.0 resonances were attributed to the two carbons associated with an internal ether. The deduction was confirmed by the chemical shift of C-7 (δ_C_ 64.4) and C-17 (δ_C_ 70.0) to downfield in ^13^C-NMR spectra of **1** compared with C-7 (δ_C_ 49.6) and C-17 (δ_C_ 60.6) of szechenyianine A [9], the signals of which are shielded by oxygen atom. In the HMBC spectrum (Figure 2), correlations of H-5 (δ_H_ 2.41) and H-6 (δ_H_ 4.12) to C-7 (δ_C_ 64.4), and H-1 (δ_H_ 3.45) to C-17 (δ_C_ 70.0), suggested the involvement of an internal ether bond. The correlation of H-14 (δ_H_ 4.84) to the carbonyl carbon signal of the benzoyl group (δ_C_ 166.4) suggested that the benzoyl group was located at C-14. The correlations of OCH_3_ (δ_H_ 3.37) to C-1 (δ_C_ 80.3), OCH_3_ (δ_H_ 3.29) to C-6 (δ_C_ 82.2), OCH_3_ (δ_H_ 3.21) to C-8 (δ_C_ 83.3), OCH_3_ (δ_H_ 3.80) to C-16 (δ_C_ 93.2), and OCH_3_ (δ_H_ 3.29) to C-18 (δ_C_ 76.6) suggested that five methoxyl groups were linked at C-1, C-6, C-8, C-16, and C-18. The correlations of H-12 (δ_H_ 1.84, 2.24) and H-14 (δ_H_ 4.84) to C-13 (δ_C_ 74.7), and H-16 (δ_H_ 3.22) to C-15 (δ_C_ 76.4), suggested that two hydroxyl groups were linked at C-13 and C-15. Thus, the planar structure of **1** was deduced as 14-benzoyloxy-13, 15-dihydroxy-1, 6,8,16,18-pentamethoxyl-7(17)-oxide-aconitane. In the ROESY spectrum (Figure 2) of **1**, the NOE correlations of H-1/H-3, H-3/H-5, H-5/H-10, H-10/H-9, H-10/H-14, H-14/H-9, and H-9/H-6 indicated *β*-orientation of H-1, H-5, H-6, H-9, H-10, and H-14, and *α*-axial configurations of 1-OCH_3_, 6-OCH_3_, and 14-benzoyloxy. NOE correlations of H-6/H-5 and H-5/H-18 revealed *β*-orientation of H-18 and 18-OCH_3_; NOE correlations of H-17/H-7, H-15/16-OCH_3_ revealed *α*-axial orientation of H-16, H-17, and 15-OH and *β*-orientation of 16-OCH_3_, 13-OH, and 8-OCH_3_. Moreover, the NOE correlations of H-1/H-3 and H-5 and the lack of correlation between H-2 and H-5 indicated that ring A (C-1, C-2, C-3, C-4, C-5, and C-11) in **1** was in the chair conformation. Thus, Compound **1** was assigned the name (A-*c*)-14*α*-benzoyloxy-13*β*,15*α*-dihydroxy-1*α*,6*α*,8*β*,16*β*,18*β*-pentamethoxy-7(17)-oxide-aconitane.

**Figure 3:**
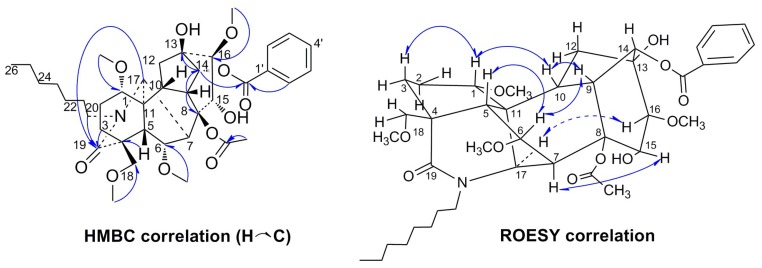
Key HMBC (H→ C) and ROESY (H↔H) correlations of Compound **2**.

Szechenyianine E (**2**) was isolated as a white amorphous powder and showed a positive reaction with Dragendorff′s reagent. Its molecular formula C_39_H_55_NO_11_ was derived from the protonated molecular ion peak at *m*/*z* 714.3840 [M + H]^+^ (calcd.714.3853) in the HR-ESI-MS spectrum. The ^1^H-NMR spectrum (Table 1) of **2** showed the presence of five aromatic proton signals due to a monosubstituted benzene at δ_H_ 8.01 (2H, d, *J* = 7.5 Hz), 7.56 (1H, t, *J* = 7.5 Hz), 7.45 (2H, t, *J* = 7.5 Hz), four OMe protons at δ_H_ 3.75 (3H, s), 3.30 (3H, s), 3.19 (3H s), and 3.12 (3H, s); one acetoxyl proton at δ_H_ 1.35 (3H, s), and one methylic proton of the hydrocarbon chain at δ_H_ 0.86 (3H, t, *J* = 6.2 Hz). The ^13^C-NMR spectrum (Table 1) displayed 39 carbon resonances. Among them, the resonances at δ_C_ 166.2, 133.6, 129.9, 129.8 (C × 2), and 128.9 (C × 2) were attributed to a benzoyloxy group; δ_C_ 61.5, 59.4, 58.0, and 55.5 were attributed to four OMe groups, δ_C_ 172.5 and 21.6 were attributed to one acetoxyl group; δ_C_ 173.3 was attributed to C=O, δ_C_ 74.3 and 79.0 were attributed to two carbons associated with the hydrocarbon chain, and δ_C_ 14.3 was attributed to one CH_3_ group. The assignments of the NMR signals associated with **2** were derived from HSQC, HMBC, and ROESY experiments. In the HMBC spectrum (Figure 3), correlations of H-14 (δ_H_ 4.89) to the carbonyl carbon signal of the benzoyl group (δ_C_ 166.4) suggested that the benzoyl group was located at C-14. Correlations of OCH_3_ (δ_H_ 3.19) to C-1 (δ_C_ 82.4), OCH_3_ (δ_H_ 3.12) to C-6 (δ_C_ 83.3), OCH_3_ (δ_H_ 3.75) to C-16 (δ_C_ 90.4), and OCH_3_ (δ_H_ 3.30) to C-18 (δ_C_ 80.0) suggested that four methoxyl groups were linked at C-1, C-6, C-16, and C-18, respectively. Correlations of CH_3_ (δ_H_ 1.32) to 8-OAc (δ_C_ 172.5) suggested that one acetoxyl was linked at C-8, and correlations of H-3 (δ_H_ 2.61, 2.71), H-17 (δ_H_ 4.11), and H-20 (δ_H_ 3.04, 3.92) to C-19 (δ_C_ 173.3) suggested that C=O was linked at C-19. Correlations of H-20 (δ_H_ 3.04, 3.92) to C-17 (δ_C_ 56.7) and C-21 (δ_C_ 33.7), H-21 (δ_H_ 1.51, 1.62) to C-22 (δ_C_ 25), and H-22 (δ_H_ 1.59, 1.68), H-25 (δ_H_ 1.30, 2H, m), and H-26 (δ_H_ 0.86, 3H, t) to C-24 (δ_C_ 31.9) suggested the presence of an *N*-heptyl group. Correlations of H-12 (δ_H_ 2.19, 2.80), H-14 (δ_H_ 4.89), and H-16 (δ_H_ 3.30) to C-13 (δ_C_ 74.3), and H-16 (δ_H_ 3.30) to C-15 (δC 79.0) suggested that two hydroxyl groups were linked at C-13 and C-15, respectively. This compound differed from the known compound (A-*c*)-8*β*-acetoxy-14*α*-benzoyloxy-*N*-ethyl-13*β*,15*α*-dihydroxy-1*α*,6*α*,16*β*,18*β*-tetramethoxy-19-oxo-aconitane [14] only in terms of the substituents on the N atom. According to the ROESY (Figure 3) spectrum, NOE correlations of H-6/H-5 and H-5/H-18 revealed *β*-orientation of H-18 and 18-OCH_3_, *α*-axial orientation of 6-OCH_3_; NOE correlations of H-7/H-15, H-17/H-16 revealed *α*-axial orientation of H-16, H-17, and 15-OH and *β*-orientation of 16-OCH_3_, 13-OH, and 8-OAc. Moreover, the NOE correlations of H-3/H-1/H-10/H-9/H-6/H-5 and the lack of correlation between H-2 and H-5 indicated that Ring A (C-1, C-2, C-3, C-4, C-5, and C-11) in **2** was in the chair conformation, the relative configuration of this compound was confirmed. Thus, the planar structure of **2** was assigned the name (A-*c*)-14*α*-benzoyloxy-8*β*-acetoxyl-*N*-heptyl-13*β*,15*α*-dihydroxy-1*α*,6*α*,16*β*,18*β*-tetramethoxy-19-oxo-aconitane.

**Figure 4:**
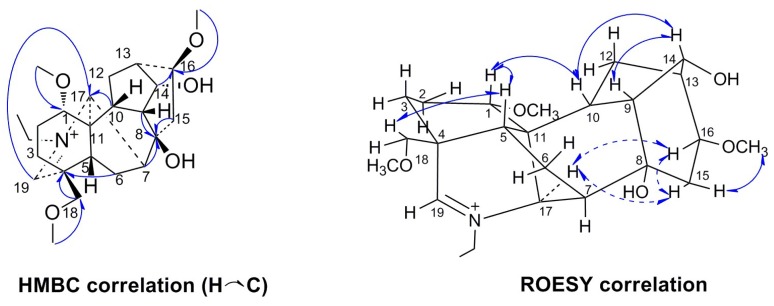
Key HMBC (H→C) and ROESY (H↔H) correlations of Compound **3**.

Szechenyianine F (**3**) was isolated as a white amorphous powder and showed a positive reaction with Dragendorff′s reagent. Its molecular formula C_24_H_38_NO_5_^+^ was derived from the ion peak at *m/z* 421.2782[M]^+^ (calcd.421.2823) in the HR-ESI-MS spectrum. The ^1^H-NMR spectrum (Table 1) of **3** showed the presence of a methine proton due to one N=CH group at δ_H_ 9.19 (1H, s), one *N*–CH_2_CH_3_ group at δ_H_ 1.51 (t, *J* =7.2 Hz), 4.01 (dq, *J* =7.2, 13.9 Hz ), and 4.42 (dq, *J* =7.2, 13.8 Hz), and three OMe resonances at δ_H_ 3.38 (3H, s), 3.36 (3H, s), and 3.16 (3H, s). The ^13^C-NMR spectrum (Table 1) displayed 24 carbon resonances. Among them, the resonances at δ_C_ 59.8, 56.9, and 56.7 were attributed to three OMe groups, δ_C_ 179.2 was attributed to one N=CH group, and δ_C_ 14.0 and 56.3 were attributed to one N–CH_2_CH_3_ group. Comparison of the NMR data of N–CH_2_CH_3_ and C-19 with those of the known compound **11** in [15] indicated the existence of the ^＋^N=CH group. The assignments of the NMR signals associated with **3** were based on HSQC, HMBC, and ROESY experiments. In the HMBC spectrum (Figure 4), correlations of H-5 (δ_H_ 2.46) and H-17 (δ_H_ 3.79) to C-19 (δ_C_ 179.2) suggested that C-19 was involved in the N=CH group. Correlations of OCH_3_ (δ_H_ 3.18) to C-1 (δ_C_ 80.6), OCH_3_ (δ_H_ 3.36) to C-16 (δ_C_ 81.5) and of OCH_3_ (δ_H_ 3.38) to C-18 (δ_C_ 73.4) suggested that three methoxyl groups were linked at C-1, C-16, and C-18, respectively. Correlations of H-6 (δ_H_ 1.82, 2.20), H-7 (δ_H_2.22), H-9 (δ_H_ 2.47), and H-10 (δ_H_ 2.01) to C-8 (δ_C_ 72.3) and of H-16 (δ_H_ 3.45) to C-14 (δ_C_ 75.0) suggested that two hydroxyl groups were linked at C-8 and C-14, respectively. Thus, the planar structure of **3** was deduced as 8,14-dihydroxy-1,16,18-trimethoxy-19-en-aconitane. In the ROESY spectrum (Figure 4) of **3**, the NOE correlations of H-1/H-5, H-1/H-10, and H-10/H-14 indicated β-orientation of H-1, H-9, H-10, and H-14, and α-axial configurations of 1-OCH_3_, 14-OH. NOE correlations of H-5/H-18 indicated *β*-orientation of H-18 and 18-OCH_3_. NOE correlations of H-17/H-12, H-12/H-16, and H-15/H-16 indicated α-axial configurations of H-16, H-17, and 16-OCH_3_ and *β*-orientation of 8-OH. Thus, Compound **3** was assigned the name 8*β*,14*α*-dihydroxy-1*α*,16*β*,18*β*-trimethoxy-19-en-aconitane.

**Figure 5:**
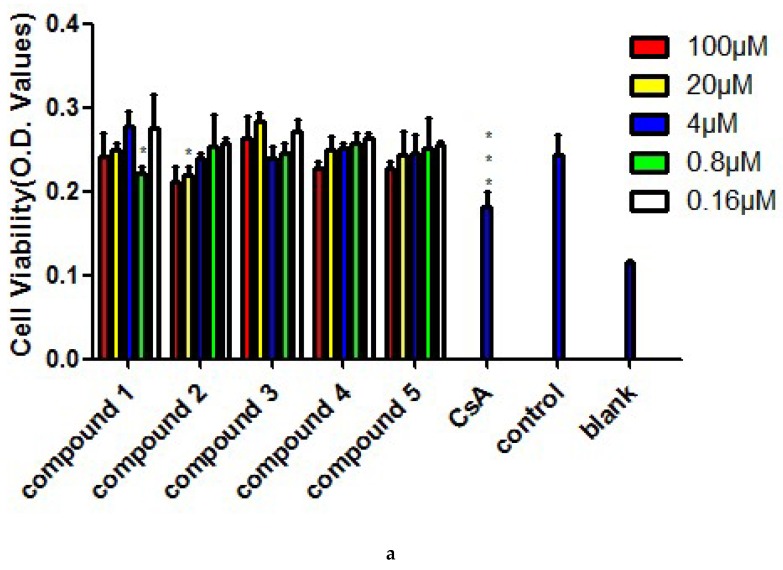

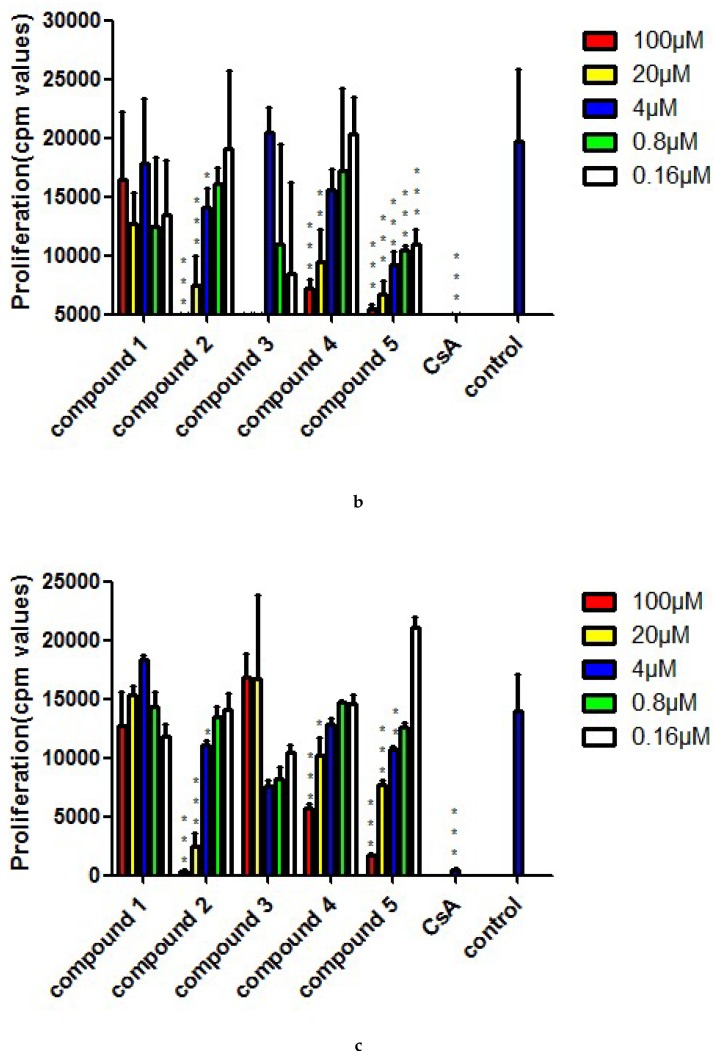
Cytotoxicity on splenocytes and inhibition on ConA- or LPS-induced splenocyte proliferation of Compounds **1**–**5**. (**a**) Cytotoxicity of Compounds **1**–**5** on BALB/c mice splenocytes. (**b**) Inhibition of Compounds **1**–**5** on ConA-induced splenocyte proliferation. (**c**) Inhibition of Compounds **1**–**5** on LPS-induced splenocyte proliferation. Results are mean ± S.D. * *p* < 0.05, ** *p* < 0.01, *** *p* < 0.001, treatment group versus control.

The roots of *A. szechenyianum* have long been used to treat rheumatic diseases, in which inflammation and suppressive immunoreaction are involved in the pathophysiological process. The immunosuppressive effects of Compounds **1**–**5** were evaluated in vitro by ConA-induced or LPS-induced splenocyte proliferation, which was suppressed in a concentration-dependent manner by **2, 4,** and **5** (Figure 5b,c), with IC_50_ values of 5.780 ± 1.12 μm, 3.151 ± 0.52 μm, and 2.644 ± 0.77 μm (ConA-induced), or 4.293 ± 3.20 μm, 3.852 ± 1.57 μm, and 2.283 ± 1.28 μm (LPS-induced), respectively. These three compounds showed low cytotoxic effect (Figure 5a), with CC_50_ values of 422.85 ± 66.4 μm, 176.35 ± 69.65 μm, and 188 ± 84.15 μm, respectively. The CC_50_/IC_50_ values of **2, 4,** and **5** suggested that these compounds are potential immunosuppressive agents. 

These three compounds had a certain immunosuppressive effects, but low cytotoxic effects compared with cyclosporin A. We will conduct further experiments in vivo using the arthritis model in rat induced by adjuvant and arthritis model in mice induced by collagen, to obtain more lead compounds to treat rheumatoid arthritis.

## 3. Materials and Methods

### 3.1. General Information

Optical rotation indices were determined in methanol on a Rudolph Autopol II digital polarimeter (Rudolph, Hackettstown, NJ, USA). ESI-MS analysis was performed on a Quatro Premier instrument (Waters, Milford, MA, USA). HR-ESI-MS spectra were recorded on an Agilent Technologies 6550 Q-TOF (Santa Clara, CA, USA). 1D- and 2D-NMR spectra were recorded on Bruker-AVANCE 400 (Bruker, Rheinstetten, Germany) and Bruker-AVANCE 600 instrument (Bruker, Rheinstetten, Germany) using TMS as an internal standard. Analytical HPLC was performed on a Waters e2695 Separations Module system coupled with a 2998 Photodiode Array Detector and an Accurasil C-18 column (4.6 mm × 250 mm, 5 μm particles, Ameritech, Chicago, IL, USA). Semipreparative HPLC was performed on a system comprising an LC-6AD pump equipped with an SPD-20A UV detector (Shimadzu, Kyoto, Japan) and an Ultimate XB-C18 (10 mm × 250 mm, 5 μm particles) or YMS-Pack-ODS-A (10mm × 250 mm, 5 μm particles) column. Silica gel was purchased from Qingdao Haiyang Chemical Group Corporation (Qingdao, China). 

### 3.2. Plant Material

The roots of *A. szechenyianum* Gay. were collected from the Xi Mountains in Gansu Province of China in July 2014 and identified by senior experimentalist Jitao Wang. A voucher specimen (herbarium No. 20140728) has been deposited in the Medicinal Plants Herbarium (MPH), Shaanxi University of Chinese Medicine, Xianyang, China. 

### 3.3. Extraction and Isolation

The air-dried and powdered underground parts of *A. szechenyianum* (5.0 kg) were extracted with 80% EtOH at 80 °C (3 × 40 L; 1.5 h). After the removal of EtOH under reduced pressure, the extract (2 L) was dispersed in water (1.5 L), adjusted to pH 0.8 with 9% HCl solution, and extracted with petroleum ether (PE). The acidic water solution was alkalized to pH 10.26 with 25% ammonia solution, extracted with CHCl_3_ three times, and evaporated under pressure to give crude alkaloids (50 g). The crude alkaloids (47 g) were loaded on a silica gel column and eluted with a gradient solvent system (PE/acetone/diethylamine, 50:1:0.1–1:1:0.1) to yield 12 fractions (Fr.1–Fr.12). Fr.3 (2.5 g) was purified by HPLC (YMC-Pack-ODS-A, 10 × 250 mm, 5 μm particles, flow rate of 1.0 mL·min^−1^) with CH_3_OH/H_2_O (83:17) as the mobile phase to obtain **1** (6 mg, t_R_ = 45 min). Fr.4 was purified by HPLC with CH_3_OH/H_2_O (75:25) as the mobile phase to obtain **4** (60 mg, t_R_ = 46 min) and **5** (40 mg, t_R_ = 58 min). Fr.7 was purified by HPLC with CH_3_OH/H_2_O (65:35) as the mobile phase to obtain **2** (7 mg, t_R_ = 50 min) and **3** (7 mg, t_R_ = 56 min). More details of the spectra are provided in the Supplementary Material.

*(A-c)-14α-benzoyloxy-8β-acetoxyl-13β,15α-dihydroxy-1α,6α,8β,16β,18β-pentamethoxyl-7(17)–oxide*-*aconitane* (szechenyianine D): A white amorphous powder, [α${]}_{D}^{23.1}$-9.3 (c 0.043, MeOH), IR (KBr) ν_max_: 3495, 2914, 1719, 1277, 1099, 1031 and 712 cm^−1^; ^1^H-NMR (400 MHz, CDCl_3_) and ^13^C-NMR (100 MHz, CDCl_3_) spectral data, see Table 1; *m*/*z* 590.2979 [M + H]^+^ (calcd. 590.2965) for C_31_H_43_NO_10_.

*(A-c)-14α-benzoyloxy-8β-acetoxy-N-nonyl-13β,15α-dihydroxy-1α,6α,16β,18β-tetramethoxy-19-oxo-aconitane* (szechenyianine E): A white amorphous powder, [α${]}_{D}^{23.0}$ + 12.1 (c 0.033, MeOH), IR (KBr) ν_max_: 3471, 2933, 2822, 1717, 1453, 1278, 1098, and 712 cm^−1^; ^1^H-NMR (600 MHz, CDCl_3_) and ^13^C-NMR (150 MHz, CDCl_3_) spectral data, see Table 1; *m*/*z* 714.3840 [M + H] ^+^ (calcd.714.3853) for C_39_H_55_NO_11_.

*8β,14α-dihydroxy-1α,16β,18β-trimethoxy-19-en-aconitane* (szechenyianine F): A white amorphous powder, [α${]}_{D}^{22.9}$-17.4 (c 0.013, MeOH), IR (KBr) ν_max_: 3381, 2933, 1630, 1455, 1376, 1096, and 1030 cm^−1^; ^1^H-NMR (400 MHz, CDCl_3_) and ^13^C-NMR (100 MHz, CDCl_3_) spectral data, see Table 1; *m*/*z* 421.2782 [M]^+^ (calcd.421. 2823) for C_24_H_38_NO_5_^+^.

### 3.4. MTT Assay

Splenocytes (4 × 10^5^ cells/well) were incubated in triplicate at 37 °C in a humidified incubator with 5% CO_2_ and 95% air. The assay was performed in a 96-well format, and different concentration of Compounds **1**–**5** (0.16–100 μm) and CsA（2μm）were added. The cells cultured with media alone were used as controls. Approximately 48 h later, 20 μL of 3-(4,5-dimethylthiazol-2-yl)-2,5-diphenyltetrazolium bromide (5 mg/mL, Sigma) was added to each well. The plates were then incubated for another 5 h. Approximately 150 μL of DMSO (Sigma) was then added to each well. Optical density was measured at 570 nm (BioTek, PowWave XS2, VT, USA). The IC_50_ and CC_50_ values were calculated according to the dose curves generated by plotting the percentage of viable cells against the test concentration on a logarithmic scale by using SPSS 15.0. CsA (Cyclosporine A, Sigma, Chicago, IL, USA) was used as a positive control. 

### 3.5. ConA- and LPS-Induced Assay

Splenocytes (4 × 10^5^ cells/well), different concentration of Compounds **1**–**5** (0.16–100 μm), and CsA（2 μm）in 96-well plates at 37 °C in a 5% CO_2_ atmosphere were cultured in triplicate for 48 h using ConA (2 μg/mL, Sigma) or LPS (1 μg/mL, Sigma). The cells cultured with media alone were used as controls. The cells were pulsed at 0.25 μCi/well of [^3^H]-thymidine for 8 h before the end of the culture period and then harvested onto glass fiber filters. [^3^H]-thymidine incorporation was measured using a beta scintillation counter (MicroBeta Trilux, PerkinElmer Life Sciences, Boston, MA, USA).

## Figures and Tables

**Table 1:** ^1^H-NMR and ^13^C-NMR spectral data for Compounds **1**–**5**.

NO.	1		2		3		4	5
	δ_C_	δ_H_ (*J* in Hz)	δ_C_	δ_H_ (*J* in Hz)	δ_C_	δ_H_ (*J* in Hz)	δ_C_	δ_C_
1	80.3	3.45 (d, 7.6)	82.4	3.07 (d, 8.3)	80.6	3.40 (m)	82.8	82.8
2	29.6	1.22 (m, H-2a)	25.3	1.43 (m, H-2a)	20.6	1.40 (m, H-2a)	33.6	33.6
		2.41 (m, H-2b)		1.87 (m, H-2b)		1.72 (m, H-2b)		
3	29.9	1.22 (m, H-3a)	33.4	2.61 (m, H-3a)	25.5	1.94 (m)	72.0	71.9
		1.40 (m, H-3b)		2.71 (m, H-3b)				
4	43.5		37.8		48.6		43.3	43.2
5	42.3	2.41 (d, 6.7)	47.6	2.29 (d, 6.7)	38.4	2.46 (m)	46.2	45.9
6	82.2	4.12 (d,6.7)	83.3	4.04 (d, 6.7)	24.6	1.82 (m, H-6a)2.20 (m, H-6b)	83.6	83.7
7	64.4	3.32 (s)	50.9	2.63 (s)	45.1	2.22 (m)	45.4	43.3
8	83.3		91.3		72.3		82.6	82.5
9	44.5	2.59 (t, 5.8)	43.5	2.84 (t, 5.8)	53.8	2.47 (m)	42.7	45.4
10	40.8	2.26 (m)	41.1	2.19 (m)	38.3	2.01 (m)	41.7	41.6
11	50.9		50.0		51.2		50.8	50.8
12	35.5	2.24 (m, H-12a)	35.6	2.80 (m, H-12a)	27.8	2.04 (m, H-12a)1.26 (m, H-12b)	36.5	36.5
		1.84 (m, H-12b)		2.19 (m, H-12b)				
13	74.7		74.3		43.3	1.96 (m)	75.0	75.0
14	78.8	4.84 (d, 5.8)	78.8	4.89 (d, 5.8)	75.0	4.21 (t, 4.9)	79.7	79.8
15	76.4	4.65 (d, 5.4)	79.0	4.49 (dd, 2.9, 5.4)	39.5	2.27 (m, H-15a)	78.0	78.7
						2.40 (m, H-15b)		
16	93.2	3.22 (d, 5.4)	90.4	3.30 (d, 5.4)	81.5	3.45 (m)	93.6	93.6
17	70.0	4.29 (s)	56.7	4.11 (s)	68.1	3.79 (s)	62.7	61.4
18	76.6	3.54(d, 8.2, H-18a)	80.0	3.78(d,8.2,H-18a)	73.4	3.71 (2H, m)	77.2	77.2
		3.46 (d,8.2, H-18b)		3.06(d,8.2,H-18b)				
19	50.3	3.62(d,11.9,H-19a)	173.3		179.2	9.19 (s)	49.2	49.2
		3.72(d,11.9,H-19b)						
20			45.5	3.04 (m, H-20a)				
				3.92 (m, H-20b)				
21			33.7	1.51 (m, H-21a)				
				1.62 (m, H-21b)				
22			25.0	1.59 (m, H-22a)				
				1.68 (m, H-22b)				
23			29.6	1.30 (m, H-23a)				
				1.23 (m, H-23b)				
24			31.9	1.30 (m, H-24a)				
				2.26 (m, H-24b)				
25			22.9	1.30 (2H, m)				
26			14.3	0.86 (3H, t,6.2)				
8-OAc			172.5					
			21.6	1.35 (s)				
8-OCH_2_CH_3_								57.4
8-OCH_2_CH_3_								15.5
1-OCH_3_	55.4	3.37 (s)	55.5	3.19 (s)	56.7	3.16 (s)	56.1	56.1
6-OCH_3_	59.3	3.29 (s)	58.0	3.12 (s)			59.4	58.8
8-OCH_3_	50.7	3.21 (s)					50.1	
16-OCH_3_	62.2	3.80 (s)	61.5	3.75 (s)	56.9	3.36 (s)	61.4	62.6
18-OCH_3_	59.0	3.29 (s)	59.4	3.30 (s)	59.8	3.38 (s)	59.3	59.3
N-CH_2_CH_3_					56.3	4.01 (dq, 13.9, 7.2)	47.6	47.6
						4.42 (dq, 13.9, 7.2)		
N-CH_2_CH_3_					14.0	1.51 (t, 7.2)	13.6	13.5
ArC=O	166.4		166.2				166.5	166.4
ArC-1′	130.0		129.9				130.4	130.6
3′, 5′	128.7	7.44 (t, 7.4)	128.9	7.45 (t, 7.5)			128.6	128.6
2′, 6′	130.0	8.00 (d, 7.4)	129.8	8.01 (d, 7.5)			129.9	129.9
4′	133.4	7.54 (t, 7.4)	133.6	7.56 (t, 7.5)			133.1	133.1

δ in CDCl_3_, in ppm from TMS; coupling constants (*J*) in Hz; ^1^H-NMR at 400 MHz and ^13^C-NMR at 100 MHz for Compounds **1, 3, 4**, and **5**, and ^1^H-NMR at 600 MHz and ^13^C-NMR at 150 MHz for Compound **2**.

## Supplementary Materials

The spectra of Compounds **1**–**3** are available online at http://www.mdpi.com/1420-3049/23/5/1108/s1.
