# Supplementary material for: C19-Norditerpenoid Alkaloids from Aconitum szechenyianum
Source: Molecules. 2018 May 8;23(5):1108. doi: 10.3390/molecules23051108 (PMC6100137; doi:10.3390/molecules23051108)
Supplement: Supplementary file 1 [file molecules-23-01108-s001.pdf]

Supplementary Data

C19-Norditerpenoid Alkaloids from *Aconitum szechenyianum*

Bei Song [1,2,†], Bingliang Jin [3,†], Yuze Li [1,†], Fei Wang [4], Yifu Yang [3], Yuwen Cui [5], Xiaomei Song [2], Zhenggang Yue [2,*] and Jianli Liu [1,*]

[1] The College of Life Sciences, Northwest University, Xi'an 710069, China; songbei168@126.com (B.S.); lyz1990yeah@163.com (Y.L.)

[2] Shaanxi Collaborative Innovation Center of Chinese Medicinal Resource Industrialization, School of Pharmacy, Shaanxi University of Chinese Medicine, Xianyang 712046, China; songxiaom@126.com (X.S.)

[3] Experiment Center for Science and Technology, Shanghai University of Traditional Chinese Medicine, Shanghai 201203, China; jin872459317@126.com (B.J.); yangyifu@mail.shcnc.ac.cn (Y.Y.)

[4] Shaanxi Institute for Food and Drug Control, Xi'an 710065, China; wf88-88@163.com (F.W.)

[5] Department of Pharmacy, Xi'an Medical University, Xi'an 710021, China; polaris_101025@163.com (Y.C.)

* Correspondence: jlliu@nwu.edu.cn (J.L.); liuxingjian1981@163.com (Z.Y.); Tel.: +86-136-0929-8392 (J.L.); +86-029-3818-2209 (Z.Y.)

† These authors contribute equally to this work.

List of Content

No.	Content	Page
1	**Figure S1.** The IR spectrum of **1** (in KBr)	S3
2	**Figure S2.** The HR-ESI-MS spectrum of **1** (in MeOH)	S4
3	**Figure S3.** The ^{1}H NMR spectrum of **1** (in $CDCl_3$)	S5
4	**Figure S4.** The ^{13}C NMR spectrum of **1** (in $CDCl_3$)	S6
5	**Figure S5.** The HSQC spectrum of **1** (in $CDCl_3$)	S7
6	**Figure S6.** The HMBC spectrum of **1** (in $CDCl_3$)	S8
7	**Figure S7.** The ROESY spectrum of **1** (in $CDCl_3$)	S9
8	**Figure S8.** The IR spectrum of **2** (in KBr)	S10
9	**Figure S9.** The HR-ESI-MS spectrum of **2** (in MeOH)	S11
10	**Figure S10.** The ^{1}H NMR spectrum of **2** (in $CDCl_3$)	S12
11	**Figure S11.** The ^{13}C NMR spectrum of **2** (in $CDCl_3$)	S13
12	**Figure S12.** The HSQC spectrum of **2** (in $CDCl_3$)	S14
13	**Figure S13.** The HMBC spectrum of **2** (in $CDCl_3$)	S15
14	**Figure S14.** The ROESY spectrum of **2** (in $CDCl_3$)	S16
15	**Figure S15.** The IR spectrum of **3** (in KBr)	S17
16	**Figure S16.** The HR-ESI-MS spectrum of **3** (in MeOH)	S18
17	**Figure S17.** The ^{1}H NMR spectrum of **3** (in $CDCl_3$)	S19
18	**Figure S18.** The ^{13}C NMR spectrum of **3** (in $CDCl_3$)	S20
19	**Figure S19.** The HSQC spectrum of **3** (in $CDCl_3$)	S21
20	**Figure S20.** The HMBC spectrum of **3** (in $CDCl_3$)	S22
21	**Figure S21.** The ROESY spectrum of **3** (in $CDCl_3$)	S23

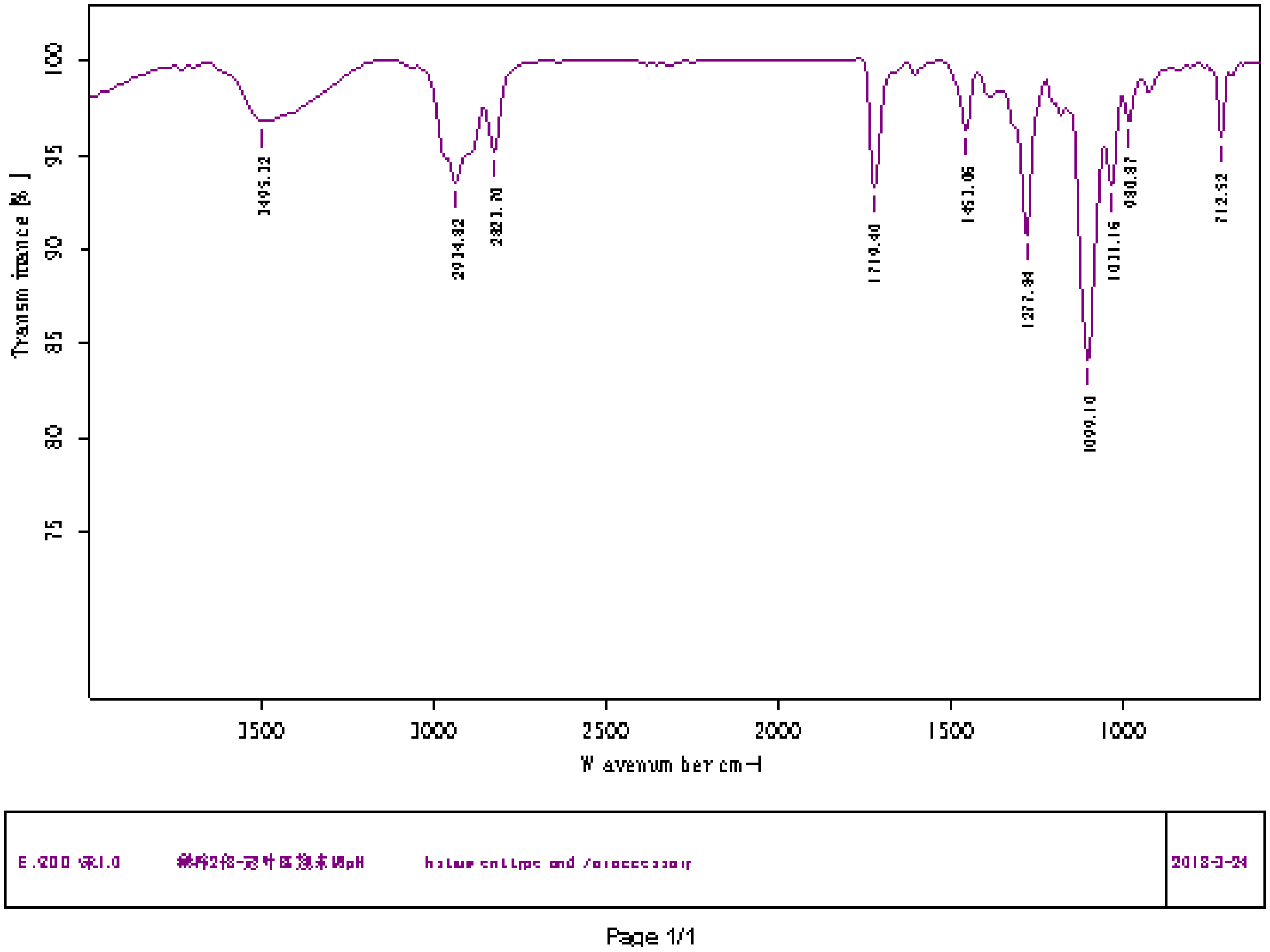

Figure S1. The IR spectrum of 1 (in KBr).

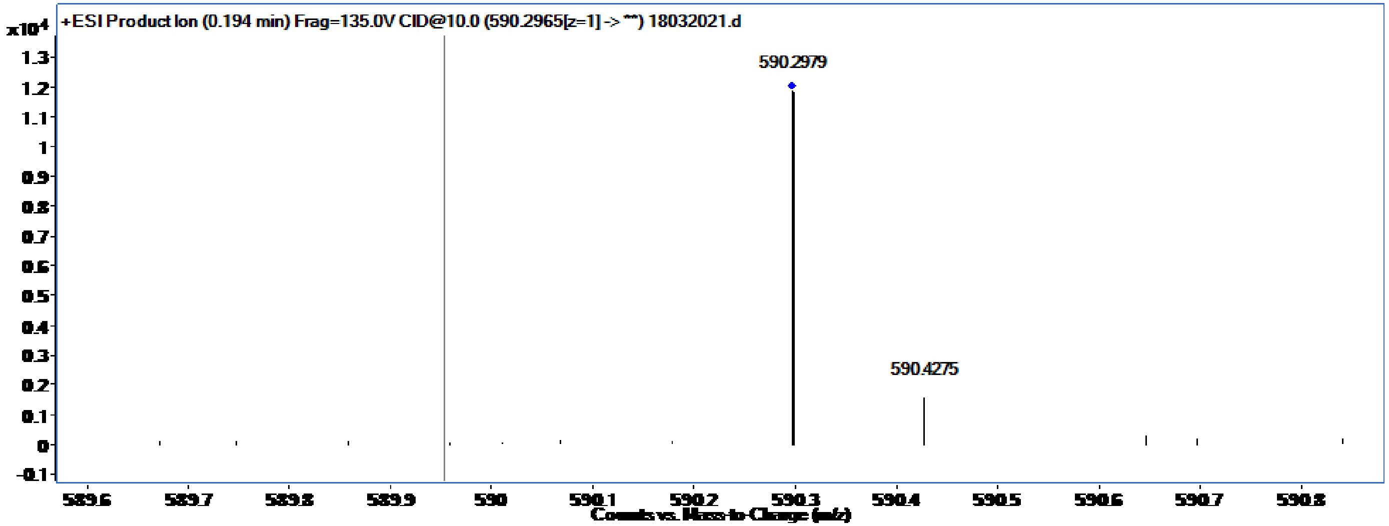

Figure S2. The HR-ESI-MS spectrum of 1(in MeOH).

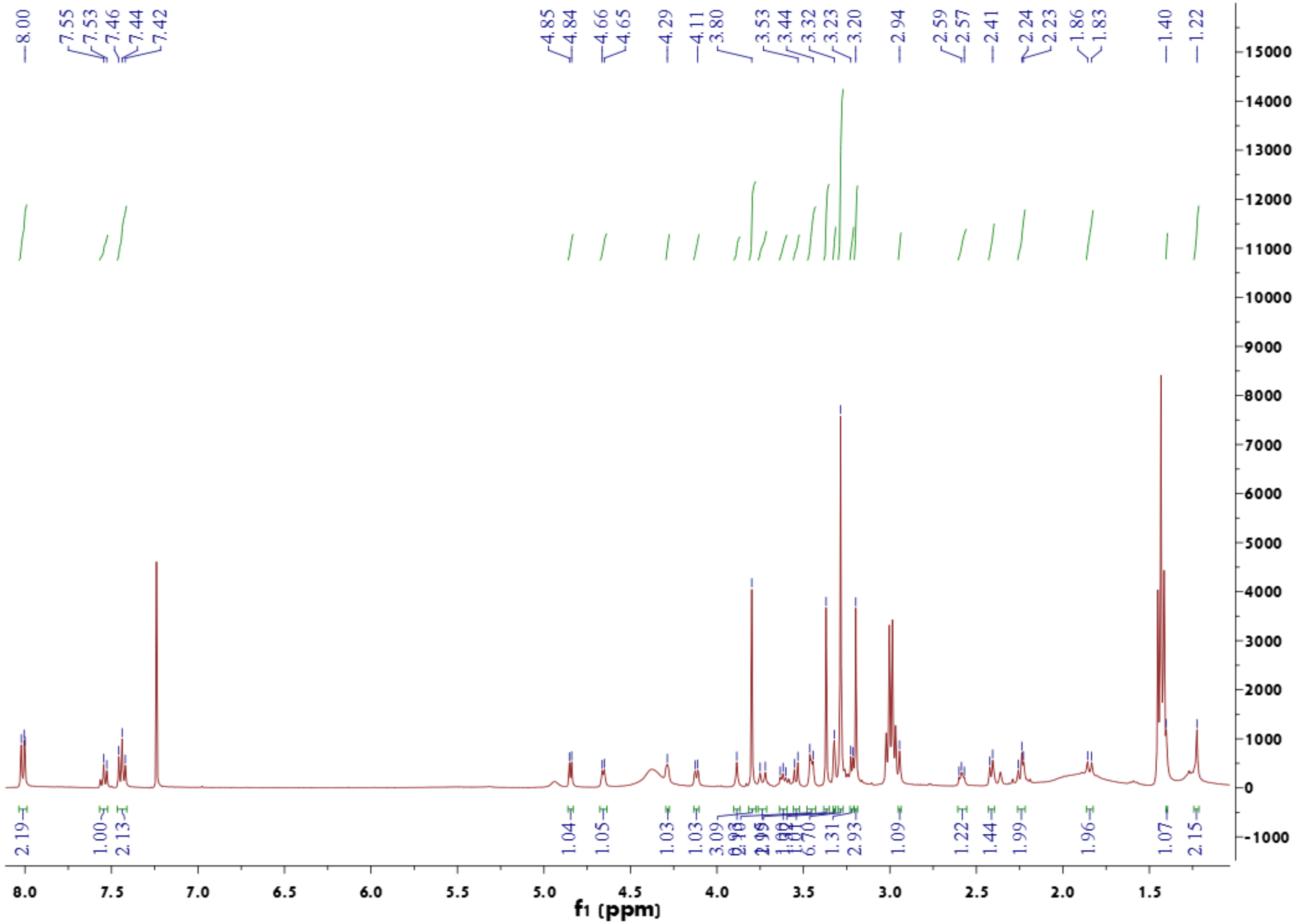

Figure S3. **The ^{1}H-NMR spectrum at 400 MHz of 1 (in $CDCl_3$).**

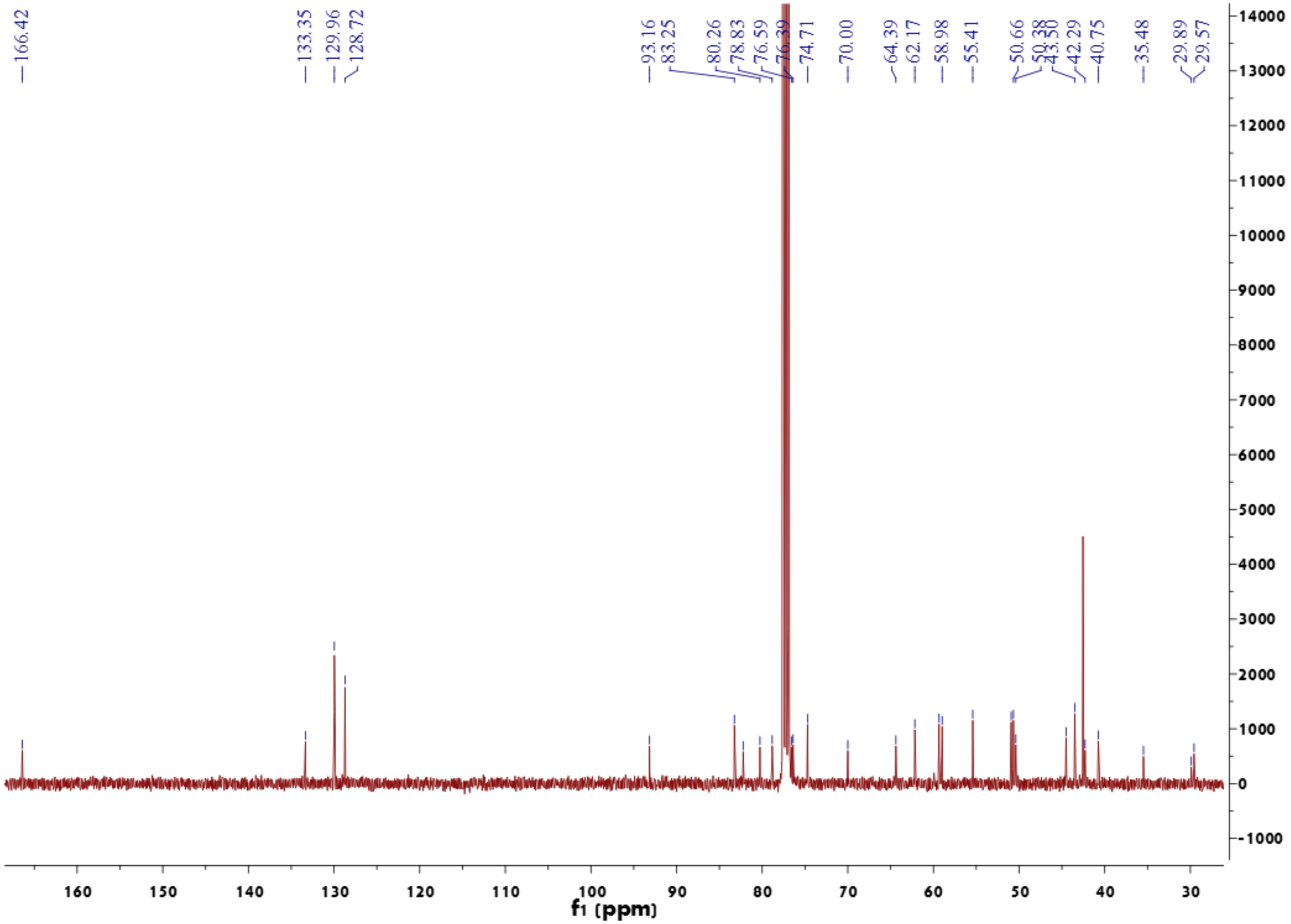

Figure S4. The ^{13}C-NMR spectrum at 100 MHz of 1 (in $CDCl_3$).

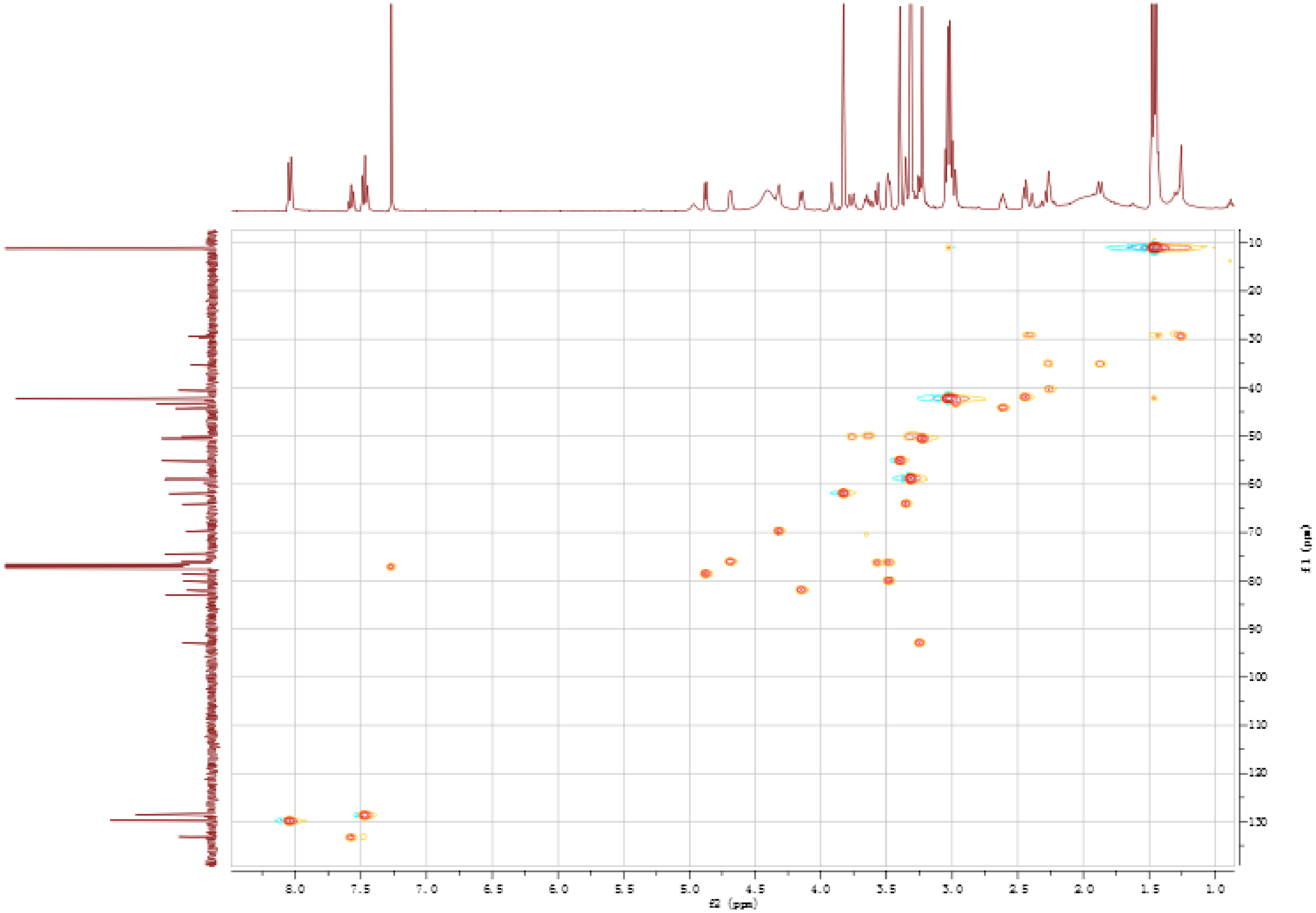

Figure S5. The HSQC spectrum of 1 (in $CDCl_3$).

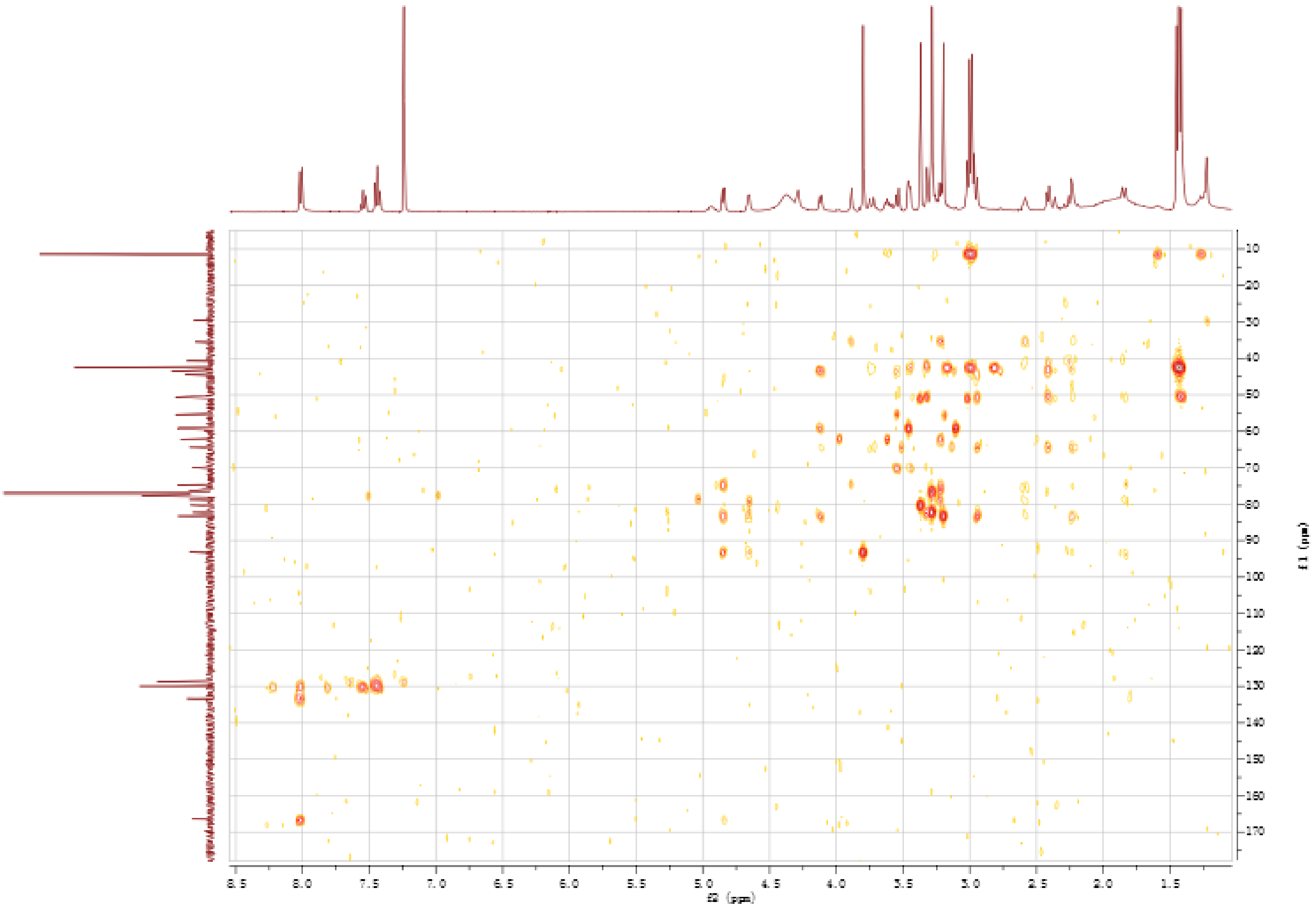

Figure S6. The HMBC spectrum of 1 (in $CDCl_3$).

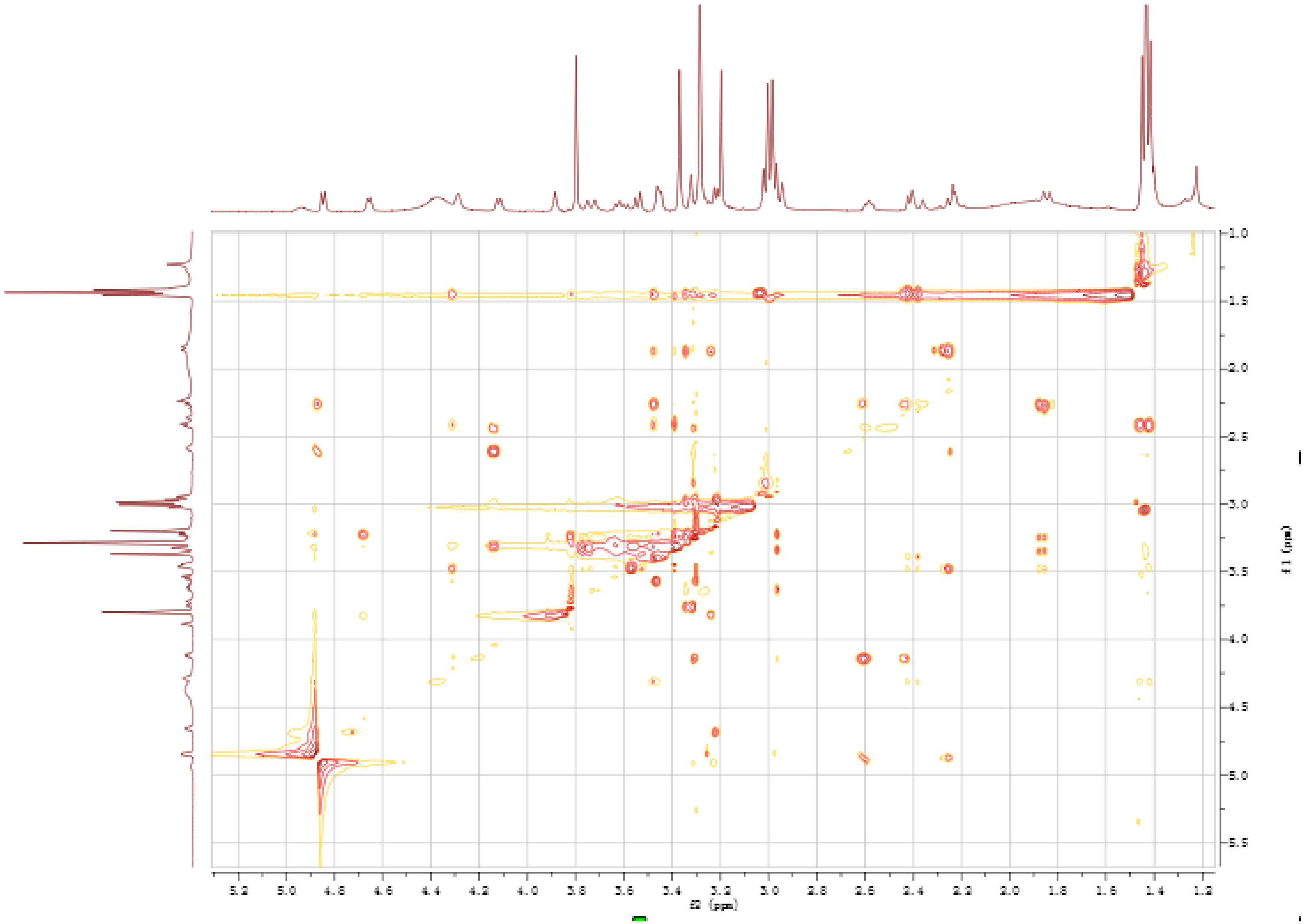

Figure S7. The ROESY spectrum of 1 (in $CDCl_3$)

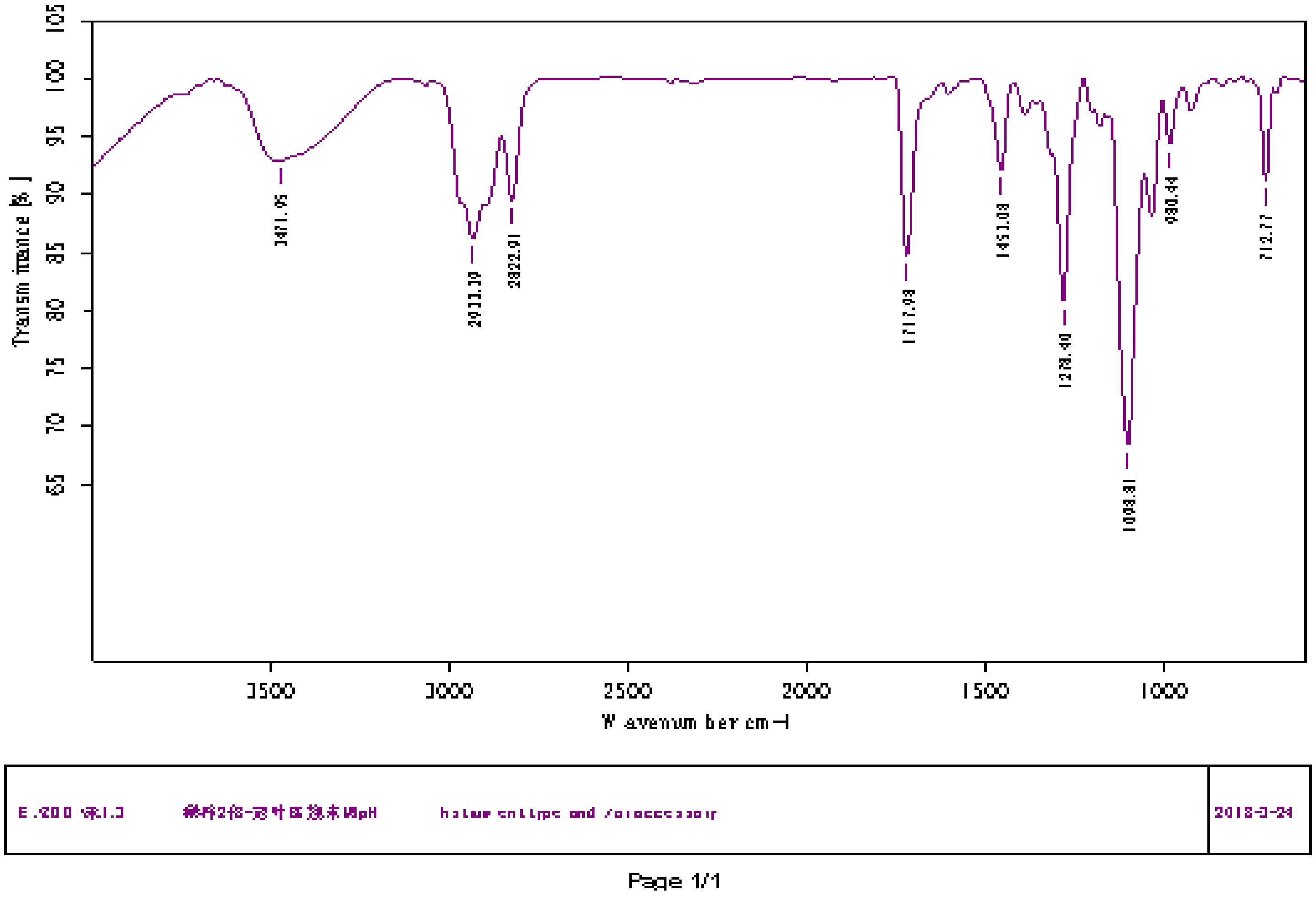

Figure S8. The IR spectrum of 2 (in KBr).

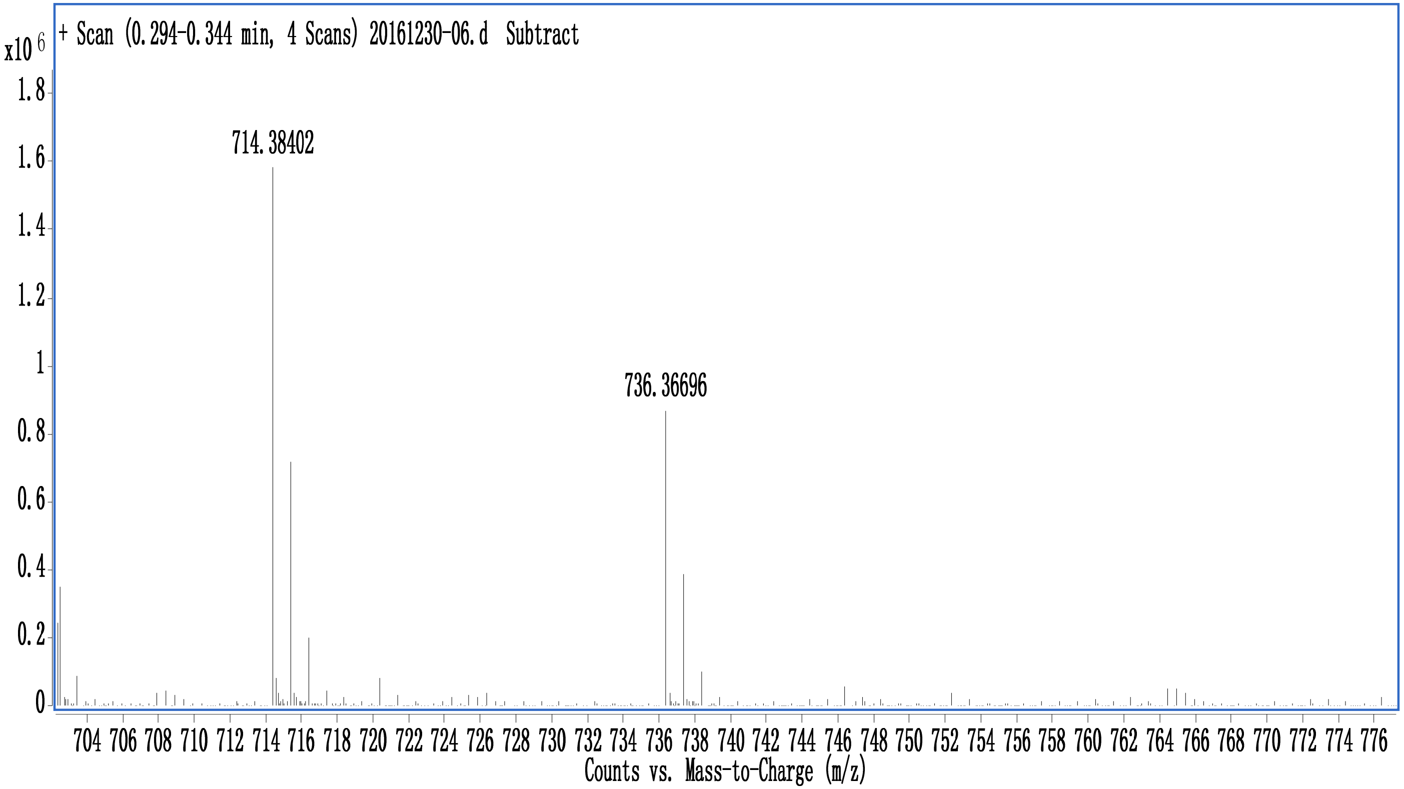

Figure S9. The HR-ESI-MS spectrum of 2(in MeOH).

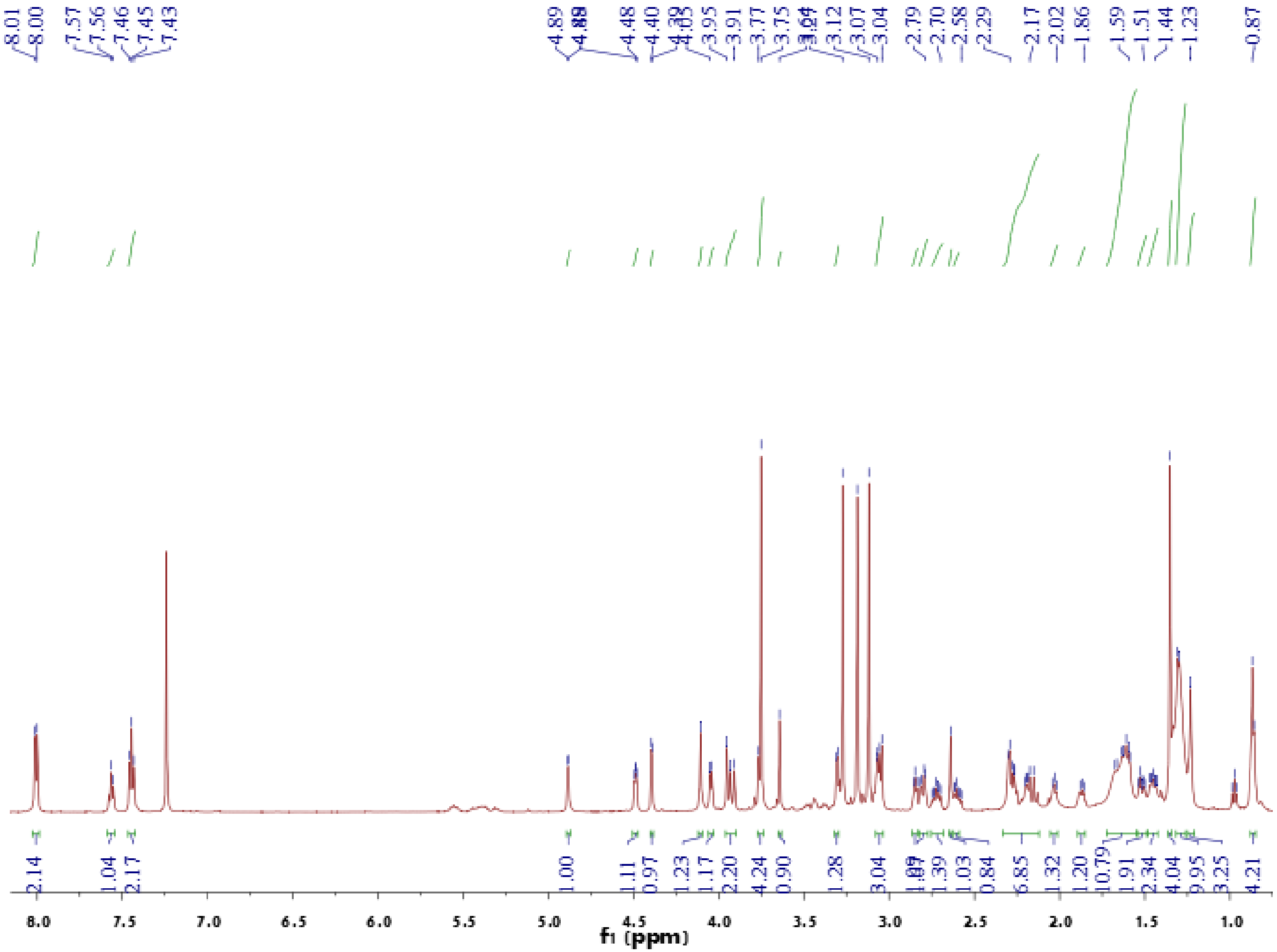

Figure S10. The ^{1}H-NMR spectrum at 600 MHz of 2 (in $CDCl_3$).

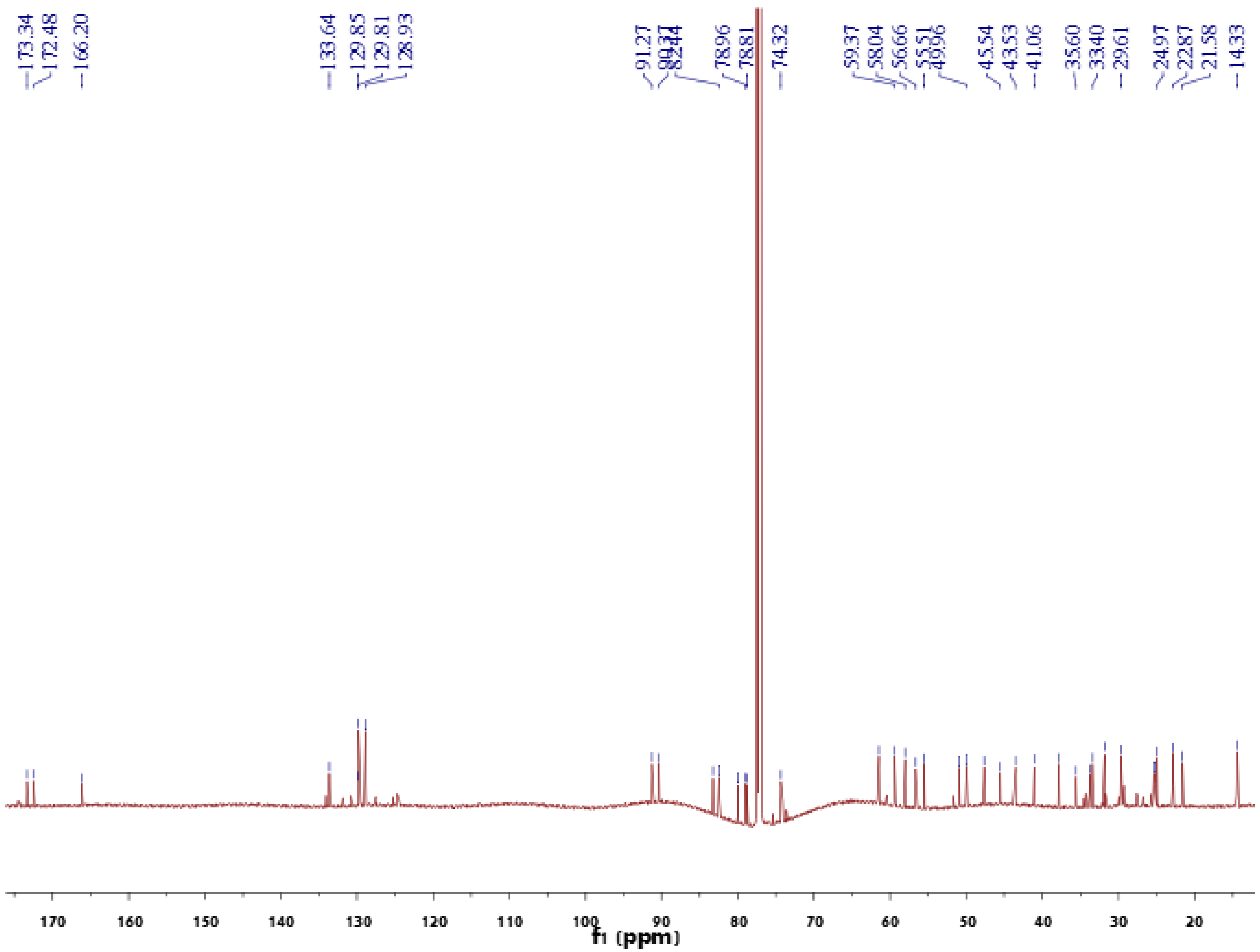

Figure S11. The ^{13}C-NMR spectrum at 150 MHz of 2 (in $CDCl_3$)

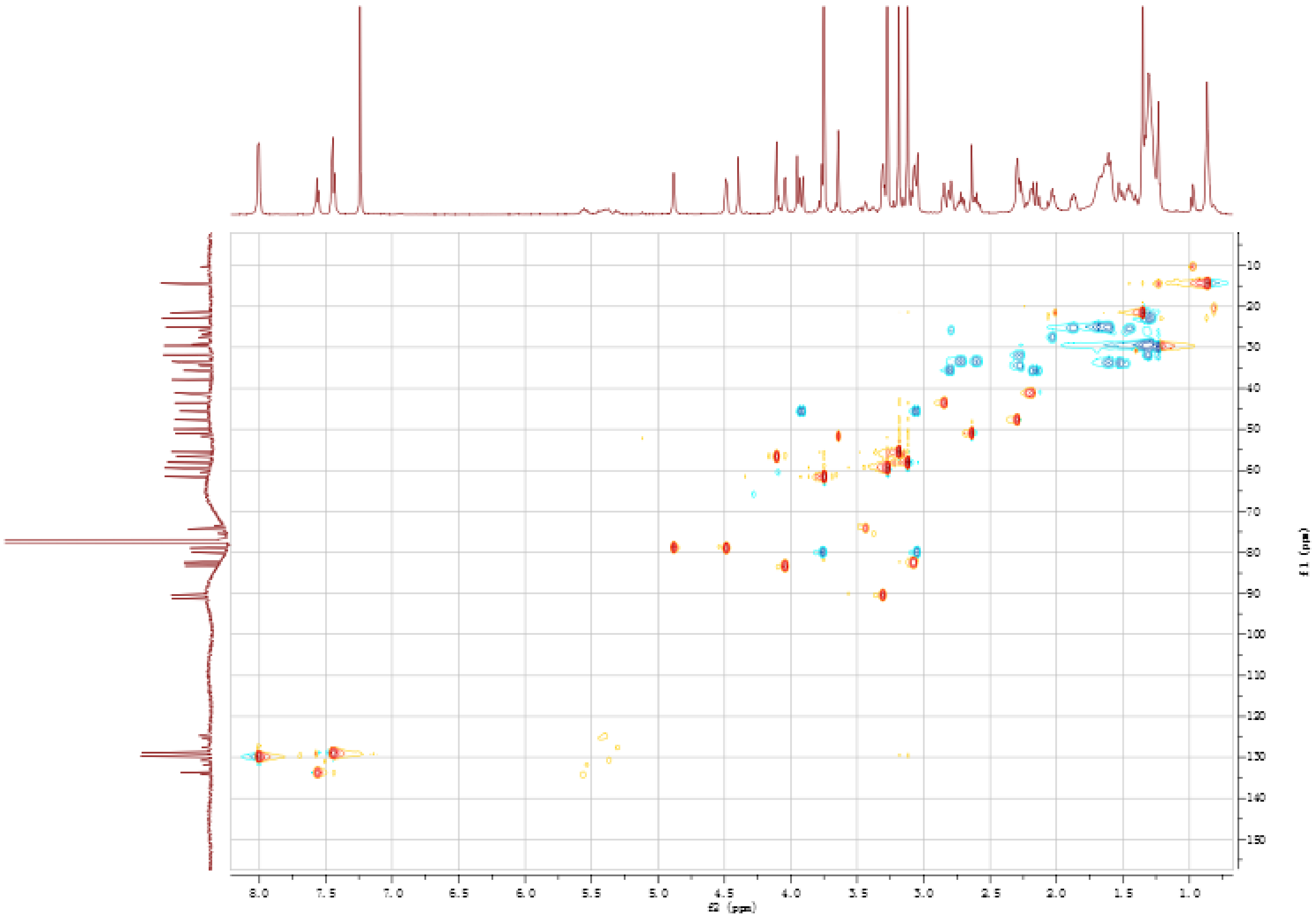

Figure S12. The HSQC spectrum of 2 (in $CDCl_3$).

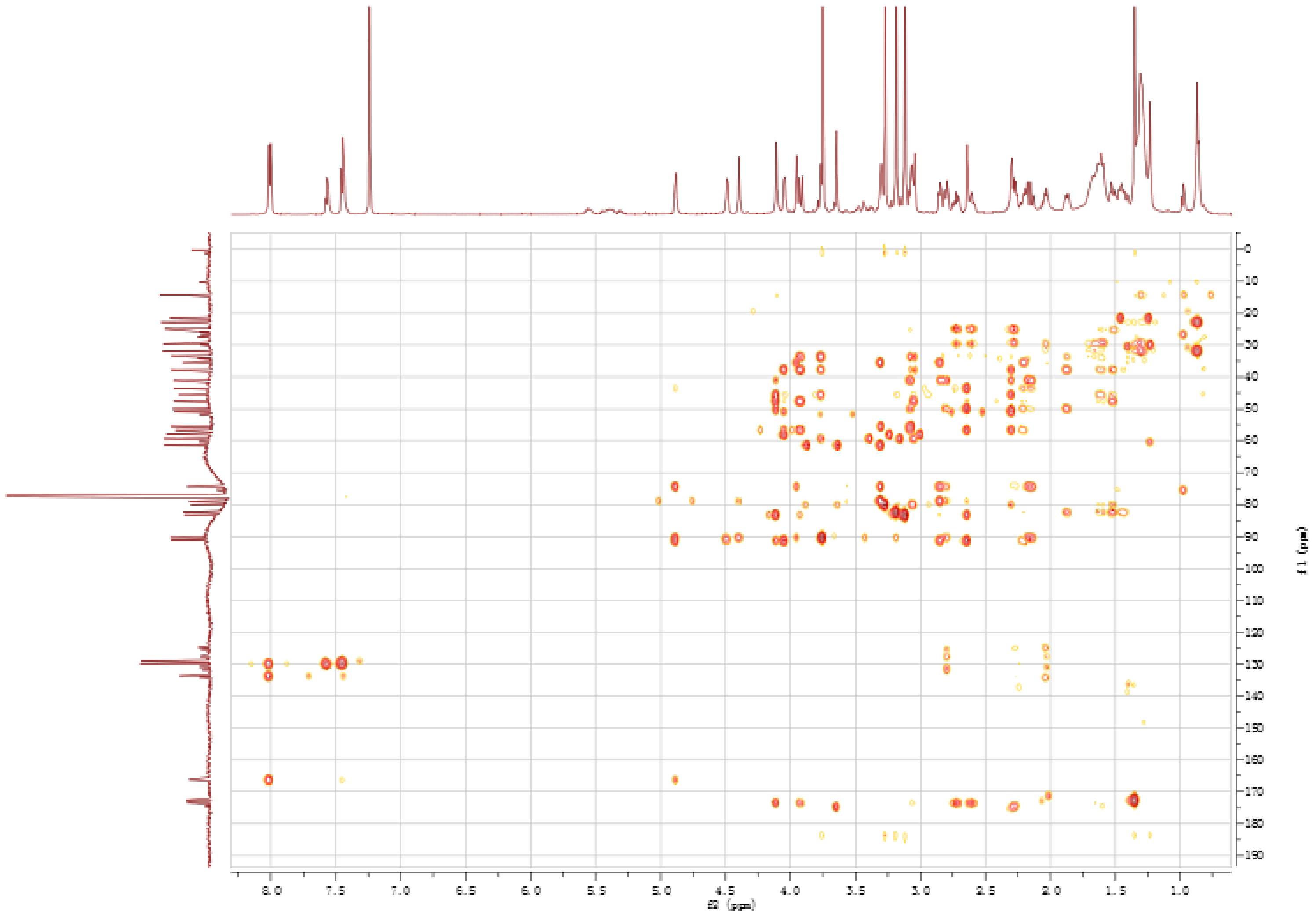

f2 (ppm)
f1 (ppm)

Figure S13. The HMBC spectrum of 2 (in $CDCl_3$).

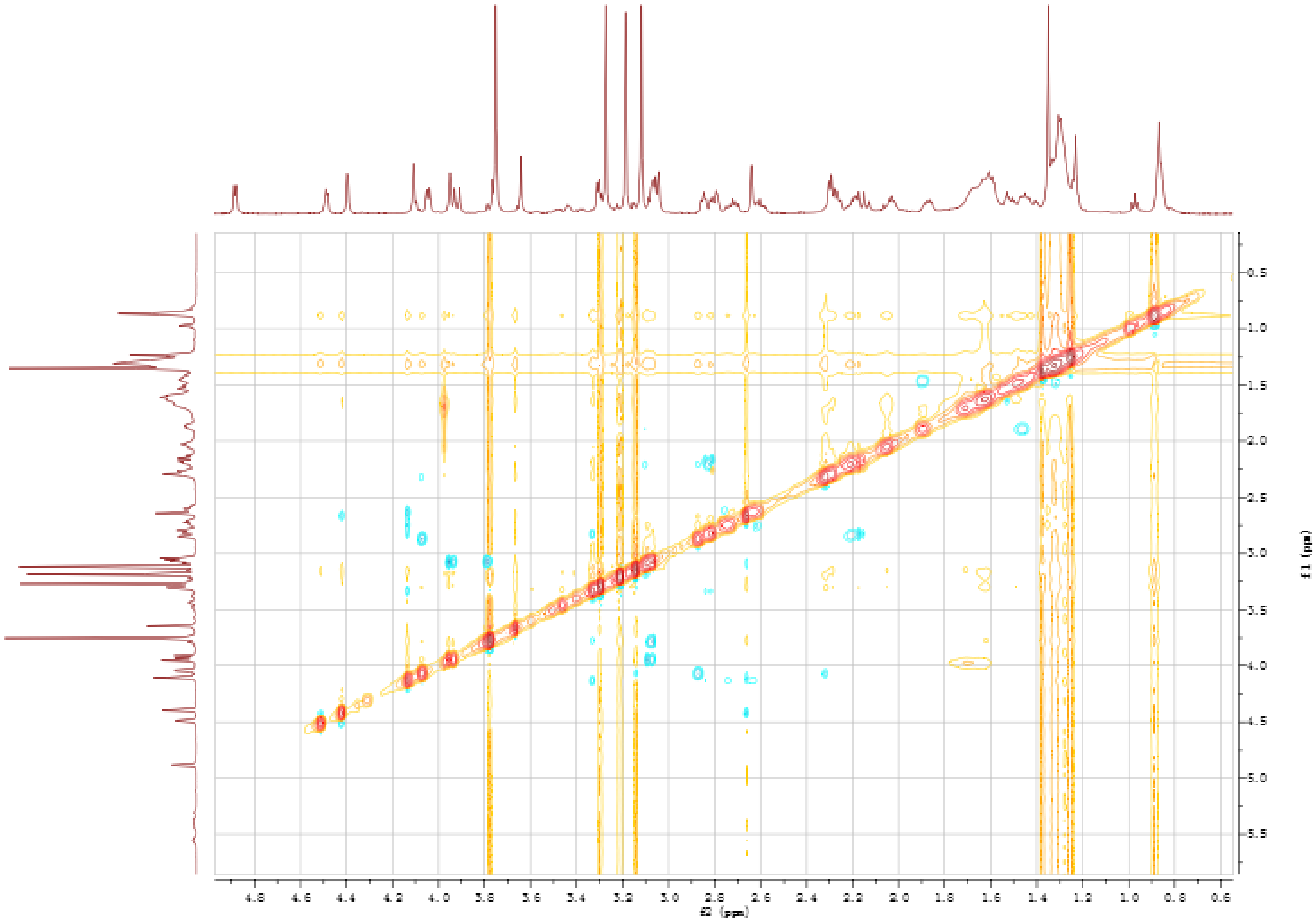

Figure S14. The ROESY spectrum of 2 (in $CDCl_3$).

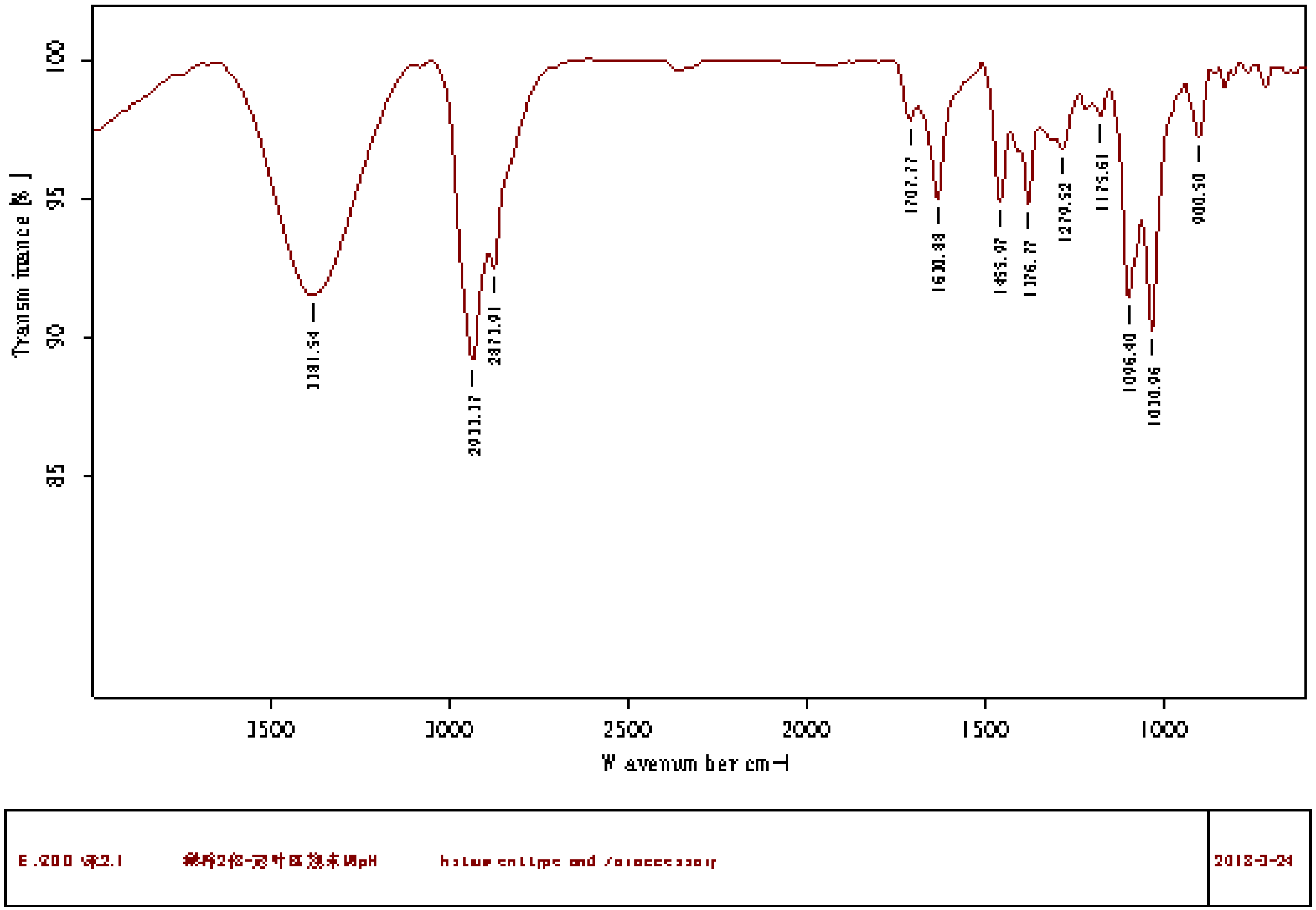

Transmittance [%]
100
95
90
85
3381.54
2933.37
2873.91
1707.77
1630.88
1455.07
1376.77
1270.52
1175.61
1096.40
1030.96
900.50
3500
3000
2500
2000
1500
1000
Wavenumber cm-1
2018-3-24

Page 1/1

Figure S15. The IR spectrum of 3 (in KBr).

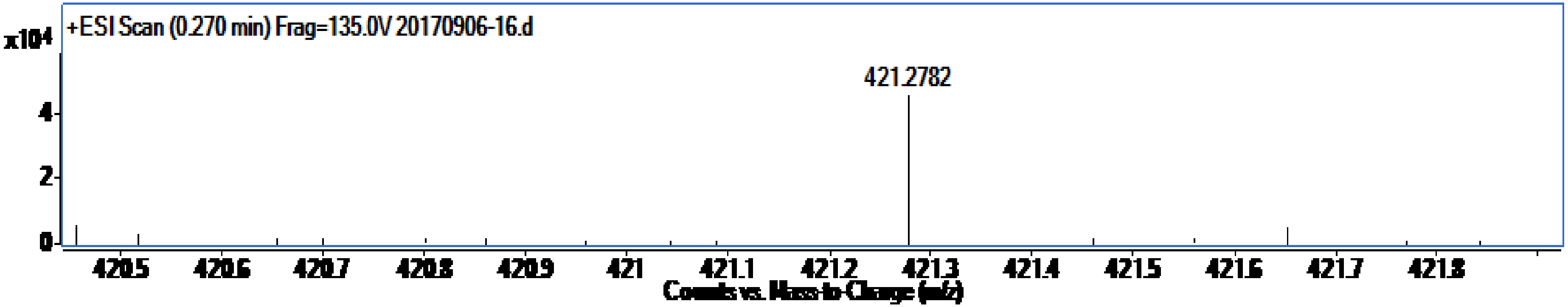

Figure S16. The HR-ESI-MS spectrum of 3(in MeOH).

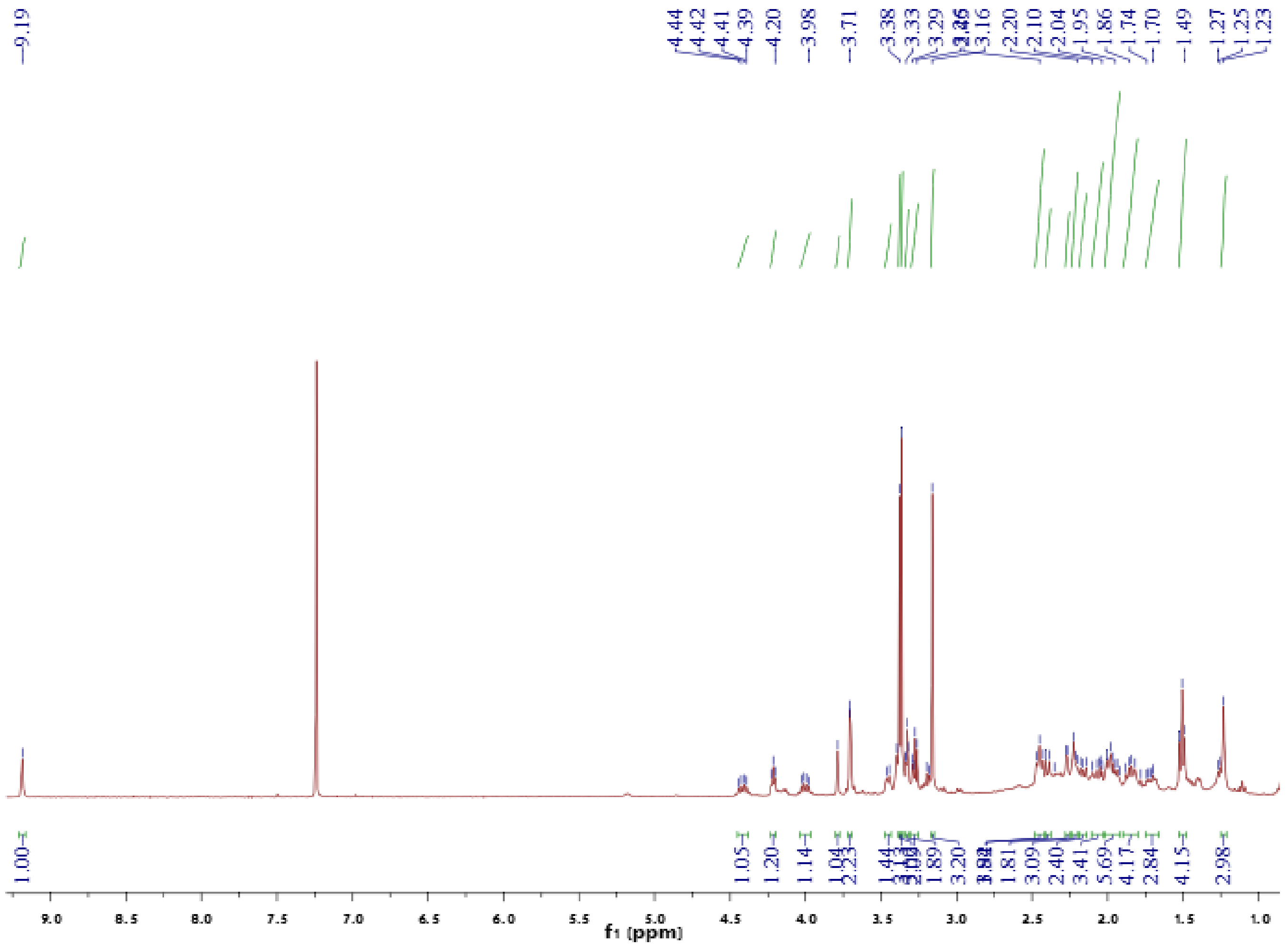

Figure S17. The ^{1}H-NMR spectrum at 400 MHz of 3 (in $CDCl_3$).

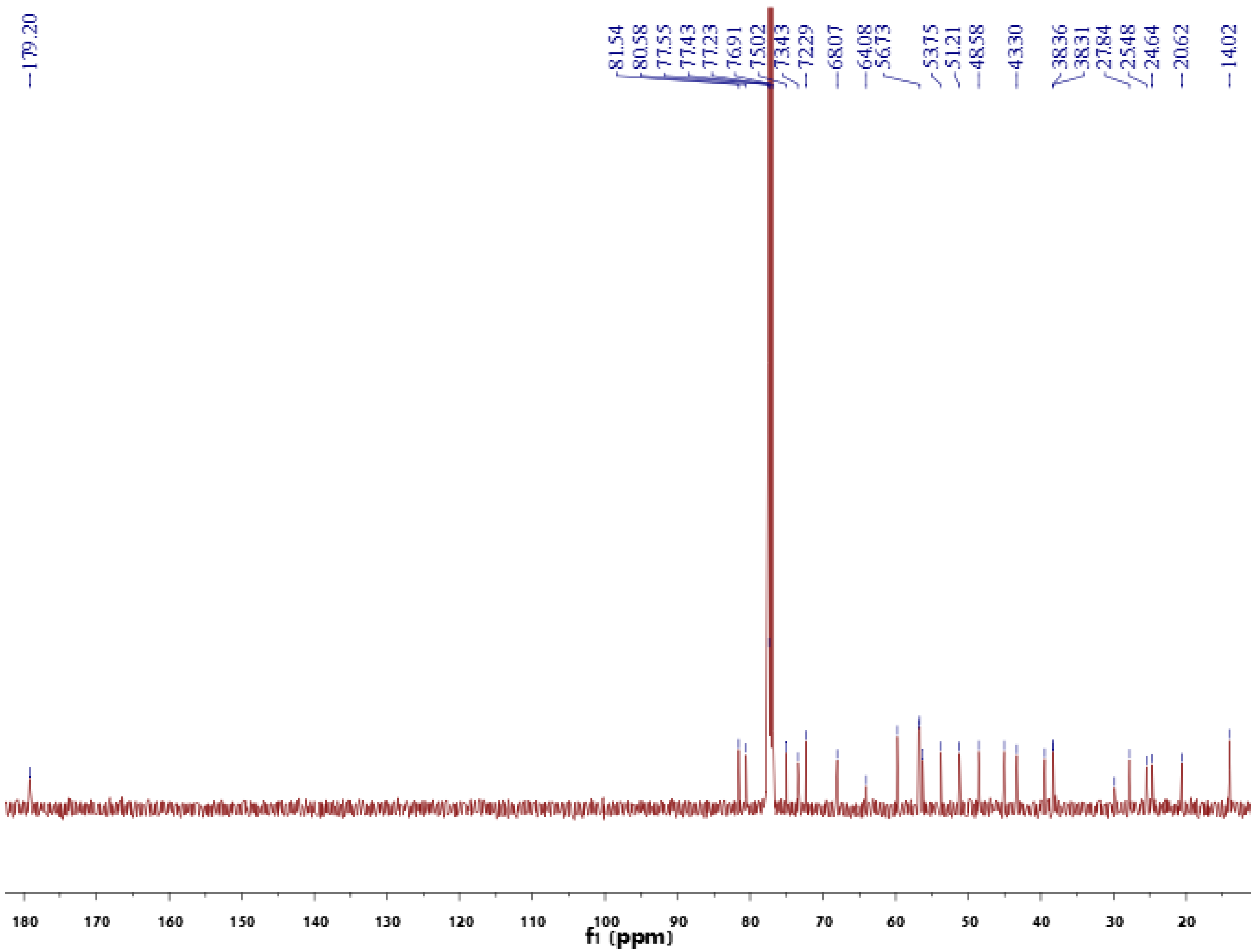

Figure S18. The ^{13}C-NMR spectrum at 100 MHz of 3 (in $CDCl_3$).

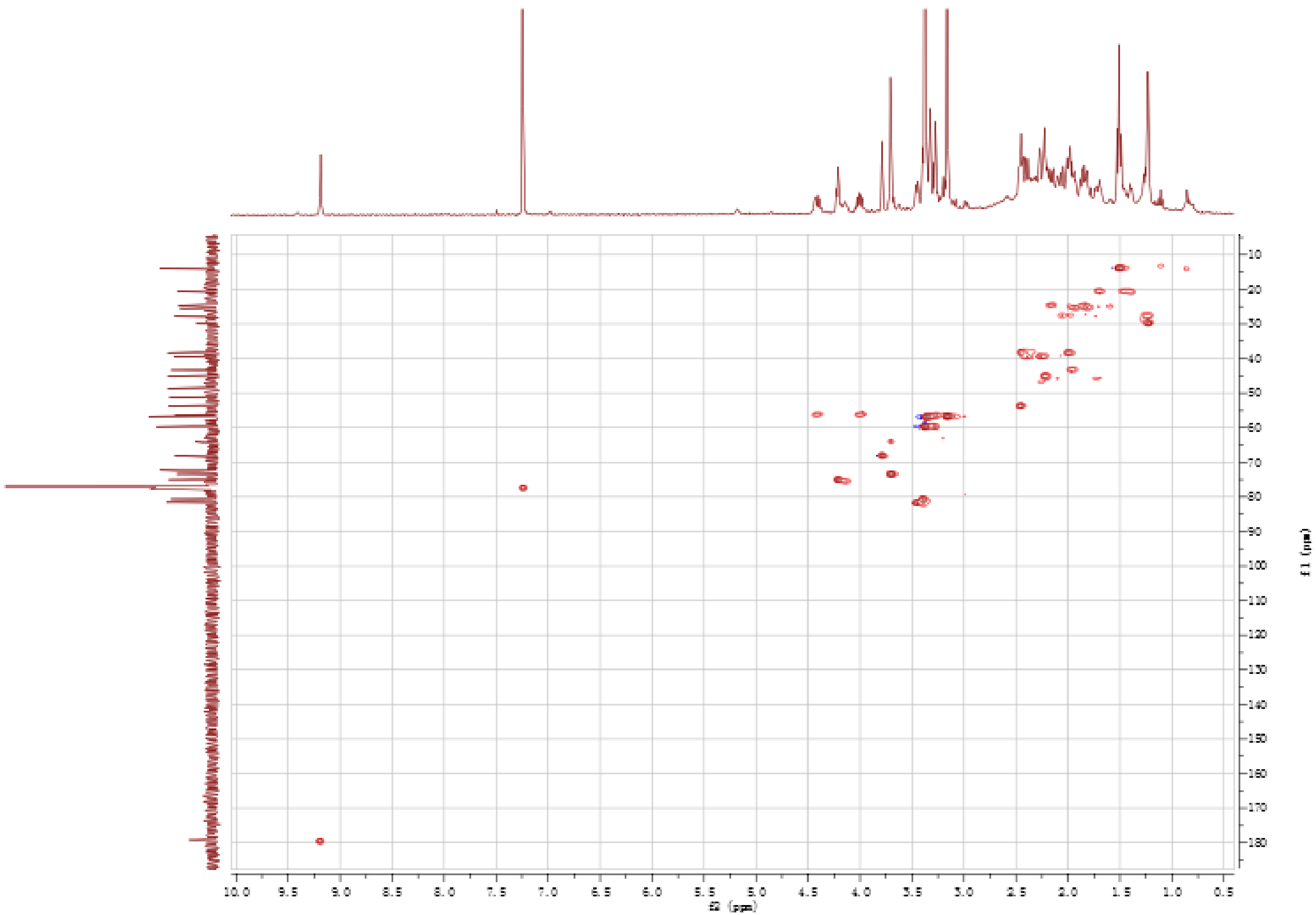

Figure S19. The HSQC spectrum of 3 (in $CDCl_3$).

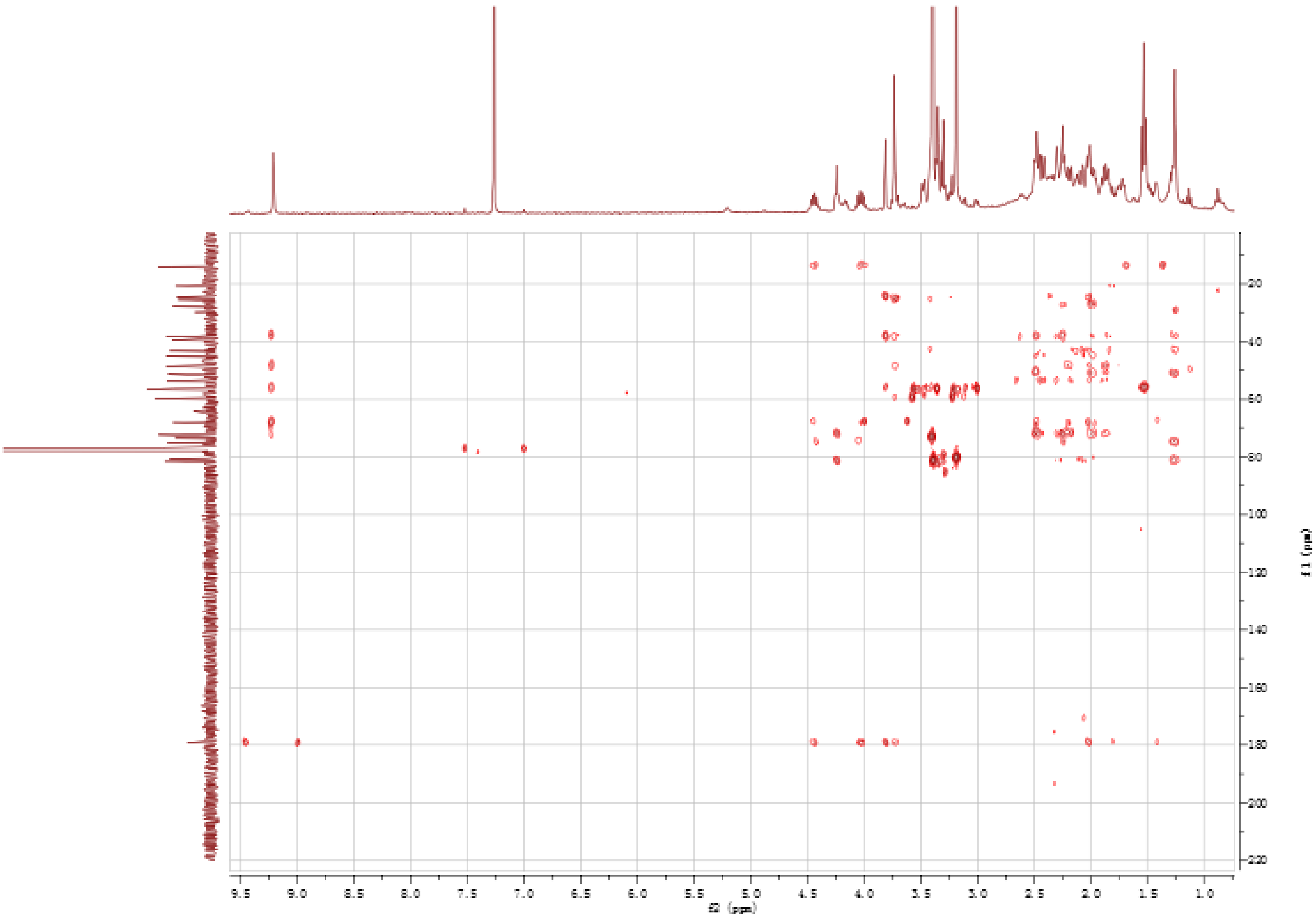

Figure S20. The HMBC spectrum of 3 (in $CDCl_3$).

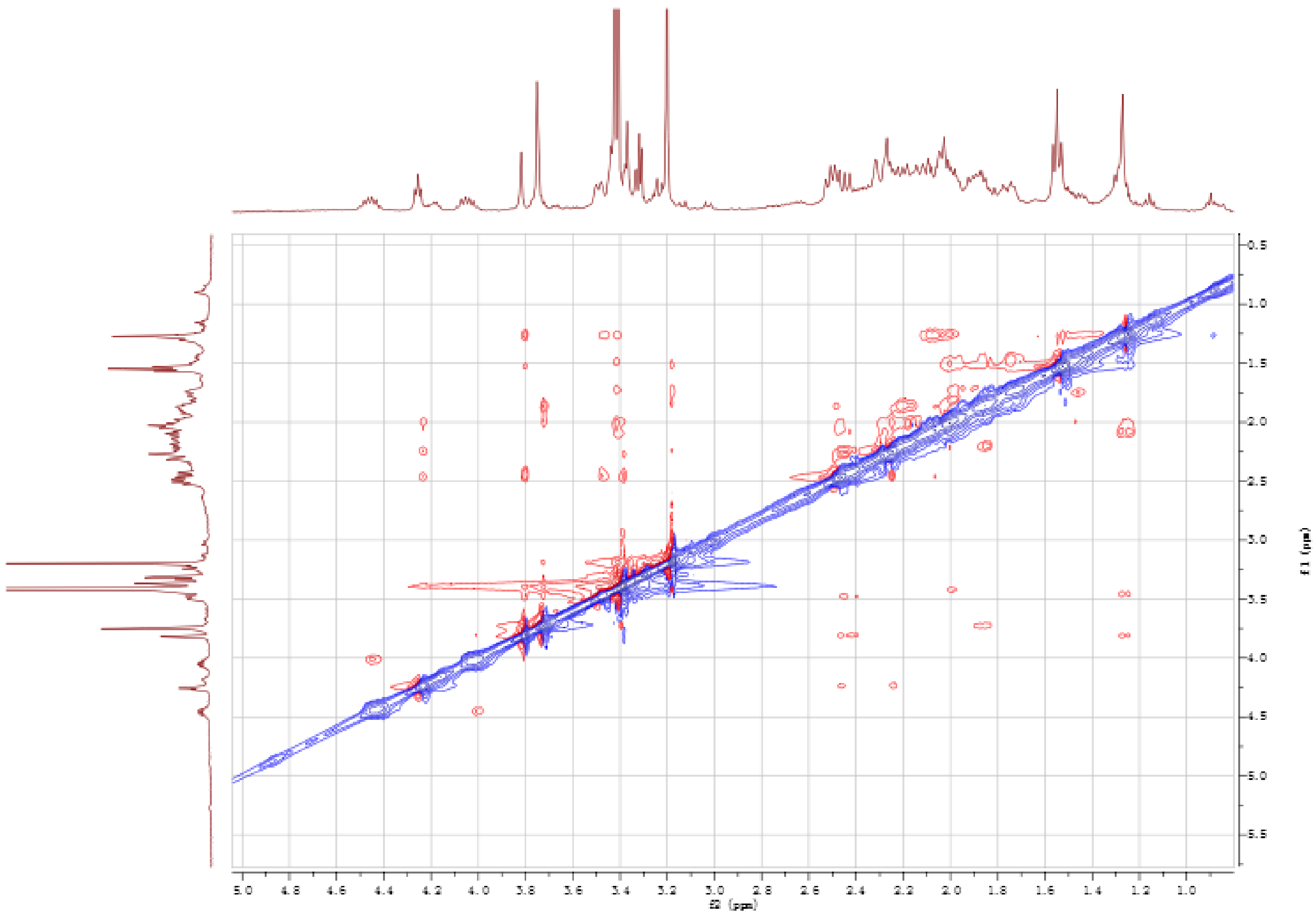

Figure S21. The ROESY spectrum of 3 (in CDCl3).
